# Some New Properties of an Active Flux Type Scheme: PamPa

**DOI:** 10.1007/s10915-026-03377-7

**Published:** 2026-07-01

**Authors:** Rémi Abgrall, Yongle Liu, Philipp Öffner

**Affiliations:** 1 Institute of Mathematics, University of Zürich, 8057 Zürich, Switzerland; 2 Institute of Mathematics, Clausthal University of Technology, D-38678 Clausthal-Zellerfeld, Germany

## Abstract

In this paper, we provide a few new properties of Active Flux (AF)/Point-Average-Moment PolynomiAl-interpreted (PamPa) schemes. First, we show, in full generality, that the AF/PamPa schemes can be interpreted in such a way that the discontinuous Galerkin (dG) scheme is one of their building blocks. Secondly we provide intrinsic bound preserving properties of the current variant of PamPa. This is also illustrated numerically. Last, we show, at least in one dimension, that the PamPa scheme has the summation by part (SBP) property.

## Introduction.

Since the seminal work of P.L. Roe and his students, [1–6], there has been a growing interest in the so-called Active Flux schemes, see e.g. [7–14] for hyperbolic conservation laws,

1 $$\begin{aligned} \dfrac{\partial \textbf{u}}{\partial t}+\text {div }\textbf{f}(\textbf{u})=0, \textbf{x}\in \Omega \subset \mathbb {R}^d, t\ge 0 \end{aligned}$$

subjected to initial and boundary conditions. Bold letters will be used for vector valued functions. For example, the conserved variables will be denoted by bold letters ($\textbf{u}$) when they belong to $\mathbb {R}^p$ with $p>1$, and non bold ones when $p=1$. The flux is $\textbf{f}=(f_1, \ldots , f_d)$ is assumed to be at least $C^1$, and defined on $\mathcal {D}\subset \mathbb {R}^p$. This set is called the invariant domain, for example in the case of the Euler equations, it is defined by imposing a strictly positive density and internal energy.

These schemes evolve simultaneously the average values and point values on the boundary of elements, that can be quads, triangles and polygons in several space dimensions, and intervals in 1D. In these papers, several progress have been made: we are now able to handle non linear cases without spurious oscillations. Very high order schemes have been developed, see e.g. [12, 15]. Conformal and non conformal meshes can be used [14, 16]. Several very intriguing properties have been noticed, and in our opinion partially explained: the schemes, at least in the Cartesian version, seems to have very interesting properties for the low Mach flows [8].

What is missing is to understand these properties: this is the motivation of this paper. The present paper will not answer all the questions, but to provide some new links to more classical schemes, and to show that some facts that were known in one dimension (see e.g. [13]) are not specific to one dimensional problems.

In this paper we are interested in three things. We first show the connection of the PamPa scheme with discontinuous Galerkin (dG) schemes. In a way, the PamPa scheme is a ...continuous discontinuous Galerkin scheme. We show that for the 1D and multi D version of the scheme, for any order and any type of elements (interval, and simplex in several dimensions).

The second contribution is about bound preservation. In [14] it was noticed that the behavior of the average values is always smoother than that of the point values. In this paper, we show several intrinsic bound preserving properties on the average values.

The last contribution is about summation by parts properties of the PamPa scheme in one dimension.

During the review process, we have been made aware of the reference [17] which has a non empty overlap with our Sect. 2. This paper has been sent to ArXiv at the same time as we submitted posted this paper on ArXiv. In Sect. 2, we go above third order accuracy, or the one dimensional or Cartesian setting, however the main idea of connecting the approximation of (2) to finite element method is essentially the same.

Our scheme has of course a strong connection to the Active Flux family of schemes. Indeed, one of us has been very much inspired by P.L. Roe’s work, and in particular the references we have listed above, and also personal discussions. He also had the opportunity to listen to Franco Brezzi’s talks at the ICM in 2014. This talk was about Virtual Finite Elements (VEM), and is a subset of [18]. The connections were obvious, in particular how functions were approximated. For that reason, and because we do not use exact evolution operators, but the method of line, we decided not to use the vocable of “active flux”, but something else: our flux is inactive, and “inactive flux” would not have been very respectful. The naming of PamPa was introduced in [13], and we have sticked to it since, even if the question of invariant domain preservation is not always central.

## Reinterpretation of the PamPa Scheme.

In this section, we describe how the PamPa scheme can be reinterpreted starting from the dG method.

In a first step, we show in one dimension and several dimensions that in each element, we can rewrite the dG method using standard duality tools of linear algebra, in the case of a linear with constant coefficient hyperbolic. This is done for segments in one dimension, and simplex in two dimensions. The extension to 3D is straightforward. Using this, we show that the PamPa scheme of [10, 12, 19] is nothing more, when using a Runge-Kutta (RK) time procedure than, for each RK cycle, one step of dG followed by a projection onto globally continuous approximations. The case of non linear problems is also described, and we discuss several projections.

### Standard PamPa Scheme.

We consider a tessellation of the 1-D spatial domain $\Omega$ in non overlapping elements $I_{j+\frac{1}{2}}=[x_j,x_{j+1}]$ with uniform size $\Delta x_{j+1/2}=x_{j+1}-x_j$. For sake of simplicity, we consider the advection equation:

2 $$\begin{aligned} u_t+au_x=0 \end{aligned}$$

with $a>0$. We will write the flux as $f(u)=au$.

In the standard third-order PamPa (or the so-called generalized AF) scheme, the solution of (2) is approximated by a globally continuous finite element polynomial expansion^1^$u_{\mathrm h}$ within each element $I_{j+\frac{1}{2}}$:

3 $$\begin{aligned} u^{j+1/2}_{\mathrm h}(x)=u_j \varphi _0(\xi )+\overline{u}_{j+\frac{1}{2}}\varphi _1(\xi )+u_{j+1}\varphi _2(\xi ),\quad \xi =\frac{x-x_j}{\Delta x_{j+1/2}},\quad x\in I_{j+\frac{1}{2}}, \end{aligned}$$

where the quadratic polynomial basis functions are

$$\begin{aligned} \varphi _0=(1-\xi )(1-3\xi ),\quad \varphi _1=6\xi (1-\xi ), \quad \varphi _2=\xi (3\xi -2). \end{aligned}$$

The PamPa scheme is, for all $j\in \mathbb {Z}$: the average values evolve with

4a $$\begin{aligned} \Delta x_{j+1/2}\frac{\mathrm d\overline{u}_{j+1/2}}{\mathrm dt}+a(u_{j+1}-u_j)=0, \end{aligned}$$

and the point values with

4b $$\begin{aligned} \Delta x_{j+1/2}\frac{\mathrm d u_{j+1}}{\mathrm dt}+a\big ( 2u_j+4u_{j+1}-6\overline{u}_{j+1/2} \big )=0. \end{aligned}$$

The rational behind the relation for $u_{j+1}$ is that one can approximate the spatial derivative or $u_{\mathrm h}$ at $x=x_{j+1}$ by using the approximation of *u* either on $I_{j+1/2}$ of $I_{j+3/2}$ or combinations of the derivatives. Since $a>0$, we take the approximation using information from the downwind interval, i.e. $I_{j+1/2}$, so that

$$\begin{aligned} \dfrac{\mathrm d u^{j+1/2}_h}{\mathrm d x}(x_{j+1})=\dfrac{2u_j+4u_{j+1}-6\overline{u}_{j+1/2}}{\Delta x_{j+1/2}}. \end{aligned}$$

If *a* were negative, the relation (4b) would be replaced by

$$\begin{aligned} \Delta x_{j+3/2}\frac{\mathrm d u_{j+1}}{\mathrm dt}+a\big ( 6\overline{u}_{j+3/2}-4u_{j+1}-2u_{j+2}\big )=0 \end{aligned}$$

or equivalently by

$$\begin{aligned} \Delta x_{j+1/2}\frac{\mathrm d u_{j}}{\mathrm dt}+a\big ( 6\overline{u}_{j+1/2}-4u_{j}-2u_{j+1}\big )=0. \end{aligned}$$

Now, we reinterpret it as an dG scheme.

The mass matrix $\texttt{M}$ given by $\texttt{M}_{i,j}= \int _{x_j}^{x_{j+1}} \varphi _i(\xi ) \varphi _j(\xi ) \textrm{d} \xi$ is

5 $$\begin{aligned} \texttt{M}=\Delta x_{j+1/2}\begin{pmatrix} \frac{2}{15} &  -\frac{1}{10} &  -\frac{1}{30} \\ -\frac{1}{10} &  \frac{6}{5} &  -\frac{1}{10} \\ -\frac{1}{30} &  -\frac{1}{10} &  \frac{2}{15} \end{pmatrix} \end{aligned}$$

and its inverse is

6 $$\begin{aligned} \texttt{M}^{-1}=\frac{1}{\Delta x_{j+1/2}}\begin{pmatrix} 9 &  1 &  3 \\ 1 &  1 &  1 \\ 3 &  1 &  9 \end{pmatrix} \end{aligned}$$

in each element $I_{j+\frac{1}{2}}$. On the other hand, we have the following update procedure

7 $$\begin{aligned} \begin{aligned}&\int _{I_{j+\frac{1}{2}}}\varphi _0\big (\frac{x-x_j}{\Delta x_{j+1/2}}\big ) u_{\mathrm h}'(x)\;{\mathrm d}x=\overline{u}_{j+\frac{1}{2}}-\frac{u_j+u_{j+1}}{2},\\&\int _{I_{j+\frac{1}{2}}}\varphi _2\big (\frac{x-x_j}{\Delta x_{j+1/2}}\big ) u_{\mathrm h}'(x)\;{\mathrm d}x=u_{j+1}-u_j,\\&\int _{I_{j+\frac{1}{2}}}\varphi _1 \big (\frac{x-x_j}{\Delta x_{j+1/2}}\big )u_{\mathrm h}'(x)\;{\mathrm d}x=\frac{u_j+u_{j+1}}{2}-\overline{u}_{j+\frac{1}{2}}. \end{aligned} \end{aligned}$$

So that, we get in total

8 $$\begin{aligned} \texttt{M}\frac{\mathrm d}{\mathrm dt}\begin{pmatrix} u_j \\ \overline{u}_{j+\frac{1}{2}}\\ u_{j+1} \end{pmatrix} +a\begin{pmatrix} \overline{u}_{j+\frac{1}{2}}-\frac{u_j+u_{j+1}}{2} \\ u_{j+1}-u_j \\ \frac{u_j+u_{j+1}}{2}-\overline{u}_{j+\frac{1}{2}}\end{pmatrix}=0, \end{aligned}$$

which can be further written as, using (6):

9 $$\begin{aligned} \frac{\mathrm d}{\mathrm dt}\begin{pmatrix} u_j \\ \overline{u}_{j+\frac{1}{2}}\\ u_{j+1} \end{pmatrix} +\frac{a}{{\Delta x_{j+1/2}}}\begin{pmatrix} 6\overline{u}_{j+\frac{1}{2}}-4u_j-2u_{j+1} \\ u_{j+1}-u_j \\ 2u_j+4u_{j+1}-6\overline{u}_{j+\frac{1}{2}}\end{pmatrix}=0. \end{aligned}$$

We also note that

$$\begin{aligned} \frac{a}{\Delta x_{j+1/2}}\begin{pmatrix} 6\overline{u}_{j+\frac{1}{2}}-4u_j-2u_{j+1} \\ u_{j+1}-u_j \\ 2u_j+4u_{j+1}-6\overline{u}_{j+\frac{1}{2}}\end{pmatrix}=\begin{pmatrix} f'(u_j) \\ f(u_{j+1})-f(u_j)\\ f'(u_{j+1}) \end{pmatrix}. \end{aligned}$$

In the general case of $a\in \mathbb {R}$, we can interpret PamPa as follows:

1. Run dG as here,
2. Drop the update of $u_j$
  if $a>0$: coming from the interval $I_{j+\frac{1}{2}}$ and keep the internal DoFs computed from dG.
  If $a<0$, coming from the interval $I_{j-\frac{1}{2}}$ and keep the internal DoFs computed from dG.

This justifies the naming of "Continuous dG ": starting at each Runge-Kutta cycle by a globally continuous approximation, we first apply a standard dG procedure, taking into account the simplifications introduced by the global continuity. After this cycle, each point value degree of freedom is multi-valued: there is, a priori, one value per element that share this degree of freedom. Then we need to project back on the original space, this is done by taking into account upwinding. This procedure is generalized in several dimensions in the next sections

### High Order PamPa Scheme.

In the high order case, we take $m_i(x)=\big (\frac{x-x_j}{\Delta x_{j+1/2}}\big )^i$ in the interval $I_{j+\frac{1}{2}}$ and define the degrees of freedom (DoFs) for degree *k* (where the formal order of the scheme is $k+1$) as

10 $$\begin{aligned} u_0\approx u(x_j),\quad u_1\approx u(x_{j+1}),\quad u_{l+2}=\int _{I_{j+\frac{1}{2}}}m_{l}(x)u(x)\;{\mathrm d}x,~\text {for} ~0\le l\le k-2. \end{aligned}$$

Note that in (10), we have changed the ordering of the corresponding coefficients due to the high-order approach. We denote the linear forms as follows:

$$\begin{aligned} \langle \theta _l,u\rangle =u_l, \quad 0\le l\le k \end{aligned}$$

where $\theta _l$ is a bounded linear functional. We denote the dual basis in $\mathbb {P}^k$ by $\{\varphi _q\in \mathbb {P}^k\}$, $q=0, \ldots , k$ such that $\langle \theta _l,\varphi _q \rangle =\delta _{lq}$. In $I_{j+\frac{1}{2}}$, *u*(*x*) is approximated as

11 $$\begin{aligned} u_{\textrm{h}}(x)=\sum _{q=0}^{k}u_q\varphi _q(x). \end{aligned}$$

In the PamPa method, we first compute the point value evolution equations

12a $$\begin{aligned} \begin{aligned} \frac{\mathrm d u_0}{\mathrm dt}&+a\frac{\mathrm d u_{\textrm{h}}}{\mathrm dx}(x_j)=0,\\ \frac{\mathrm d u_1}{\mathrm dt}&+a\frac{\mathrm d u_{\textrm{h}}}{\mathrm dx}(x_{j+1})=0,\\ \end{aligned} \end{aligned}$$

and for the moments for $0\le l\le k-2$, we write

12b $$\begin{aligned} \frac{\mathrm d u_{l+2}}{\mathrm dt}+\int _{I_{j+\frac{1}{2}}}a\, m_{l}(x)\,\frac{\mathrm d u_{\textrm{h}}}{\mathrm dx}\;\mathrm dx=0. \end{aligned}$$

Using the same basis for $\mathbb {P}^k$, the dG method writes

$$\begin{aligned} \texttt{M}\frac{\mathrm d \textbf{U}}{\mathrm dt}+\textbf{F}=0 \end{aligned}$$

with $$\texttt{M}_{l\kappa }=\int _{I_{j+\frac{1}{2}}}\varphi _l(x)\varphi _\kappa (x)\;{\mathrm d}x$$ and

$$\begin{aligned} \textbf{F}_\kappa =\int _{I_{j+\frac{1}{2}}} a\varphi _\kappa (x)\frac{\mathrm du_{\textrm{h}}}{\mathrm dx}\;{\mathrm d}x. \end{aligned}$$

The question is to compare them.

For this, using Riesz theorem, we can write $\langle \theta _l,u\rangle =(\psi _l,u)$ where

$$\begin{aligned} (u,v)=\int _{I_{j+\frac{1}{2}}}u(x)v(x)\;{\mathrm d}x. \end{aligned}$$

and $\psi _l\in \mathbb {P}^k$ again. Clearly, for $l\ge 0$, $\psi _{l+2}=m_{l}$. For $l=0,1$ (corresponding to the vertices $x_j$ and $x_{j+1}$), we also write $\psi _l$ as linear combinations of the $\varphi _q$s:

$$\begin{aligned} \psi _l=\sum _{q=0}^{k}a_{lq}\varphi _q \end{aligned}$$

and from the orthogonality condition $\delta _{lp}=\langle \theta _l,\varphi _p\rangle =(\psi _l,\varphi _p)$,

$$\begin{aligned} \delta _{lp}{=(\psi _l,\varphi _p)=\sum _{q=0}^ka_{lq}\big (\varphi _q,\varphi _p\big )}=\sum _{q=0}^{k}a_{lq}\texttt{M}_{qp} \end{aligned}$$

that we interpret as saying that the inverse of $\texttt{M}$ is the matrix $(a_{lq})$.

Then we compute $\texttt{M}^{-1}\textbf{F}$ in a component-wise manner, and get

13 $$\begin{aligned} \begin{aligned} (\texttt{M}^{-1}\textbf{F})_p&=\sum _{l=0}^{k}a_{pl}F_l=\sum _{l=0}^{k}a_{pl}\int _{I_{j+\frac{1}{2}}}a\varphi _l\frac{\mathrm d u_{\textrm{h}}}{\mathrm dx}{\mathrm d}x\\&=a\; \int _{I_{j+\frac{1}{2}}}\Big (\sum _{l=0}^{k}a_{pl}\varphi _l\Big )\frac{\mathrm d u_{\textrm{h}}}{\mathrm dx}{\mathrm d}x\\&=a\;\int _{I_{j+\frac{1}{2}}}\psi _p\frac{\mathrm d u_{\textrm{h}}}{\mathrm dx}{\mathrm d}x. \end{aligned} \end{aligned}$$

The last step is to show that

$$\begin{aligned} a\int _{I_{j+\frac{1}{2}}}\psi _p\frac{\mathrm d u_{\textrm{h}}}{\mathrm dx}{\mathrm d}x=a\dfrac{\textrm{d}u_{\textrm{h}}}{\mathrm dx}(x_{j+p}) \end{aligned}$$

for $p=0,1$, i.e. the two linear forms associated with the points values. This is indeed the fact if $a\in \mathbb {R}$ is a constant and since $\frac{\mathrm d u_{\textrm{h}}}{\mathrm dx}\in \mathbb {P}^k$ if $u_{\textrm{h}}\in \mathbb {P}^k$.

This shows that we recover the PamPa that we can again interpret as

1. Run dG as here,
2. Drop the update of $u_j$
  if $a>0$: coming from the interval $I_{j+\frac{1}{2}}$ and keep the internal DoFs computed from dG.
  If $a<0$, coming from the interval $I_{j-\frac{1}{2}}$ and keep the internal DoFs computed from dG.

### PamPa for Triangles.

We consider the scalar problem $u_t+\text { div }\textbf{f}=0$ and the case of a triangle *K*. We want that the approximation space contains $\mathbb {P}^k$ for the accuracy. We denote the vertices by $\textbf{a}_i$, $i=1,2,3$ and $\lambda _i$ the barycentric coordinates of *K*. The DoFs are these vertices and the other $k-1$ Gauss–Lobatto points on the three edges, and the $k(k-1)/2$ additional moments [14]. All of these DoFs assemble the approximation space with dimension $N_{\text {DoFs}}=3+3(k-1)+k(k-1)/2=3k+k(k-1)/2$.

The dimension of $\mathbb {P}^k$ is $(k+1)(k+2)/2$. The number of Lagrange points on each edge, taking into account the vertices as Lagrange points, is 3*k*. We have

$$\begin{aligned} 3k+k(k-1)/2-3k=\frac{k(k-1)}{2}={\text {dim }\mathbb {P}^{k-2}}. \end{aligned}$$

These equalities can be rephrased as follows. We set $B_{\boldsymbol{\mu }}=c_{\boldsymbol{\mu }}\lambda _1^{\mu _1}\lambda _2^{\mu _2}\lambda _3^{\mu _3}$ the Bézier polynomials. For the sake of simplicity, we will write

$$\begin{aligned} \lambda _1^{\mu _1}\lambda _2^{\mu _2}\lambda _3^{\mu _3}={\boldsymbol{\lambda }}^{\boldsymbol{\mu }}. \end{aligned}$$

The multi-index ${\boldsymbol{\mu }}$ is ${\boldsymbol{\mu }}=(\mu _1,\mu _2,\mu _3)$. We set $\vert {\boldsymbol{\mu }}\vert =\mu _1+\mu _2+\mu _3$. The coefficients $c_{\boldsymbol{\mu }}$ are binomial coefficients so that the $B_{\boldsymbol{\mu }}$s can be seen as the terms in the development of $(\lambda _1+\lambda _2+\lambda _3)^k$. We define the two vector spaces:

$$\begin{aligned} V_1=\text {span}\{ B_{\boldsymbol{\mu }}, \vert {\boldsymbol{\mu }}\vert =k \text { and } \mu _1\mu _2\mu _3=0\} \end{aligned}$$

and

$$\begin{aligned} V_2=\text {span}\{ B_{\boldsymbol{\mu }}, \vert {\boldsymbol{\mu }}\vert =k \text { and } \mu _1\mu _2\mu _3>0\}. \end{aligned}$$

Clearly, $\mathbb {P}^k\subset V_1\bigoplus V_2$. The elements of $V_2$ vanish on the boundary of *K*. For the sake of clarity, we will denote the generator of $V_2$ with a specific notation

$$\begin{aligned} \psi _{{\boldsymbol{\mu }}}=\lambda _1\lambda _2\lambda _3 B_{{\boldsymbol{\mu }}} \end{aligned}$$

with

- if $$1\le k\le 2$$, $$B_{{\boldsymbol{\mu }}}=1$$,
- and for $k\ge 3$, $B_{{\boldsymbol{\mu }}}={\boldsymbol{\lambda }}^{{\boldsymbol{\mu }}}$ with
  $$\begin{aligned} \vert {\boldsymbol{\mu }}\vert =k-2. \end{aligned}$$

It is clear that $\text {dim }V_1=3k$ and $\text {dim }V_2=\text { dim }\mathbb {P}^{k-2}$. We define

$$\mathcal {I}_k=\left\{ \begin{array}{ll} \{{\boldsymbol{\mu }}, \vert {\boldsymbol{\mu }}\vert =k-2\}& \text { for }k>2\\ \{(0,0,0)\} & \text { for }k\le 2. \end{array} \right. $$

The cardinal of $\mathcal {I}_k$ is $\text { dim }\mathbb {P}^{k-2}$. We will often write $b=\lambda _1\lambda _2\lambda _3$.

We introduce the following polynomial spaces:

- For $$k\le 2$$, $$V=\mathbb {P}^k\bigoplus b\mathbb {R}$$,
- and for $$k>2$$, $$V=\mathbb {P}^k \bigoplus b\mathbb {P}^{k-2}$$

We introduce the following degrees of freedom:

- For the Gauss-Lobatto points $\sigma$ on the boundary of *K*,
  14a $$\begin{aligned} \langle \delta _\sigma , p\rangle =p(\sigma ).\end{aligned}$$
  The set of Gauss-Lobatto points is denoted by $\mathcal {G}$ and contains 3*k* points.
- In the interior,
  14b $$\begin{aligned} \langle m_{{\boldsymbol{\mu }}}, p\rangle =\frac{1}{\vert K\vert }\int _K {\boldsymbol{\lambda }}^{{\boldsymbol{\mu }}}(\textbf{x})p(\textbf{x})\; \mathrm d\textbf{x}\end{aligned}$$
  for $${\boldsymbol{\mu }}\in \mathcal {I}_k$$.

They are linear forms on *V*.

#### Lemma 2.1

The linear forms (14a)-(14b) are uni-solvant on *V*.

#### Proof

Let $\alpha _\sigma$, $\sigma \in \mathcal {G}$ and $\alpha _{{\boldsymbol{\mu }}'}$, ${\boldsymbol{\mu }}'\in \mathcal {I}_k$ be real numbers such that for all $p\in V$

15 $$\begin{aligned} \sum _{\sigma \in \mathcal {G}}\alpha _{\sigma } \langle \delta _\sigma ,p\rangle +\sum _{{\boldsymbol{\mu }}'\in \mathcal {I}_k}\alpha _{{\boldsymbol{\mu }}'}\langle m_{{\boldsymbol{\mu }}'},p\rangle =0. \end{aligned}$$

We first consider the Lagrange polynomials $L_\sigma$ at $\sigma$ and define

16 $$\begin{aligned} \varphi _\sigma =L_\sigma -\sum \limits _{{\boldsymbol{\mu }}\in \mathcal {I}_k} \frac{m_{{\boldsymbol{\mu }}}(L_\sigma )}{m_{{\boldsymbol{\mu }}}(\psi _{{\boldsymbol{\mu }}})}\psi _{{\boldsymbol{\mu }}} \end{aligned}$$

Since $\psi _{{\boldsymbol{\mu }}}$ vanishes on $\partial K$ and $L_\sigma$ is a Lagrange polynomial for the Gauss-Lobatto points, we have

$$\begin{aligned} \langle \delta _{\sigma '}, \varphi _{\sigma }\rangle =\delta _{\sigma }^{\sigma '}, \end{aligned}$$

and by construction, for all ${\boldsymbol{\mu }}\in \mathcal {I}_k$,

$$\begin{aligned} \langle m_{\boldsymbol{\mu }}, \varphi _{\sigma }\rangle =0. \end{aligned}$$

Using this to test (15), we see that $\alpha _\sigma =0$ for $\sigma \in \mathcal {G}$.

Then we test with $p=\psi _{\boldsymbol{\mu }}$, ${\boldsymbol{\mu }}\in \mathcal {I}_k$, that is

17 $$\begin{aligned} \sum _{{\boldsymbol{\mu }}'\in \mathcal {I}_k}\alpha _{{\boldsymbol{\mu }}'}\int _K \lambda _1\lambda _2\lambda _3 {\boldsymbol{\lambda }}^{{\boldsymbol{\mu }}'}{\boldsymbol{\lambda }}^{\boldsymbol{\mu }}\; \mathrm d\textbf{x}=0. \end{aligned}$$

The question reduces to that of the invertibility of the $\vert \mathcal {I}_k\vert \times \vert \mathcal {I}_k\vert$ symmetric matrix

$$A=\begin{pmatrix}\int _K \lambda _1\lambda _2\lambda _3 {\boldsymbol{\lambda }}^{{\boldsymbol{\mu }}'}{\boldsymbol{\lambda }}^{\boldsymbol{\mu }}\; \mathrm d\textbf{x}\end{pmatrix}.$$

Taking $X\in \mathbb {R}^{\vert \mathcal {I}_k\vert }$, we see that

$$\begin{aligned} X^TAX=\int _K \lambda _1\lambda _2\lambda _3 \varphi ^2(\textbf{x})\; \mathrm d\textbf{x}\end{aligned}$$

with

$$\begin{aligned} \varphi =\sum _{{\boldsymbol{\mu }}\in \mathcal {I}_k}X_{{\boldsymbol{\mu }}} {\boldsymbol{\lambda }}^{\boldsymbol{\mu }}. \end{aligned}$$

**Fig. 1:**
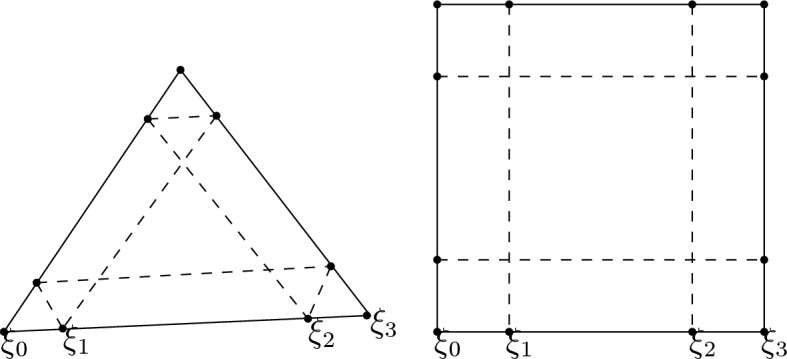
Interpolation points for the triangular and quadrangular, cubic case.

Since $\{{\boldsymbol{\lambda }}^{\boldsymbol{\mu }}\}_{{\boldsymbol{\mu }}\in \mathcal {I}_k}$ is a free family, the matrix is positive definite. Thus the forms are unisolvant. Fig. 1$\square$

The second step is to compute the dual basis of $\{\delta _\sigma , m_{\boldsymbol{\mu }}\}_{\sigma , {\boldsymbol{\mu }}}$, i.e. elements $\{\phi _\sigma , \phi _{\boldsymbol{\mu }}\}_{\sigma ,{\boldsymbol{\mu }}}$ of *V* such that for all $\sigma$,

$$\begin{aligned} \forall \sigma ', \langle \delta _\sigma , \varphi _{\sigma '}\rangle =\delta _\sigma ^{\sigma '}\text { and }\forall {\boldsymbol{\mu }}, \langle \delta _\sigma , \varphi _{\boldsymbol{\mu }}\rangle =0 \end{aligned}$$

and for all ${\boldsymbol{\mu }}$,

$$\begin{aligned} \forall \sigma , \langle m_{{\boldsymbol{\mu }}}, \varphi _{\sigma }\rangle =0 \text { and }\forall {\boldsymbol{\mu }}', \langle m_{{\boldsymbol{\mu }}}, \varphi _{{\boldsymbol{\mu }}'}\rangle =\delta _{{\boldsymbol{\mu }}}^{{\boldsymbol{\mu }}'}. \end{aligned}$$

We have the following lemma:

#### Lemma 2.2

(Dual basis) The dual basis of the forms $\delta _\sigma , m_{{\boldsymbol{\mu }}}$ consists in the elements of *V* defined by:

- Associated to the Gauss-Lobatto points, the family $\{\varphi _\sigma \}$ defined by (16).
- Associated to the moments: the elements $\varphi _{{\boldsymbol{\mu }}}$ defined by
  $$\begin{aligned} \varphi _{{\boldsymbol{\mu }}}=\lambda _1\lambda _2\lambda _3\big (\sum _{{\boldsymbol{\nu }}\in \mathcal {I}_k}a_{{\boldsymbol{\mu }}{\boldsymbol{\nu }}}\lambda ^{{\boldsymbol{\nu }}}\big ) \end{aligned}$$
  where the $\{a_{{\boldsymbol{\mu }}{\boldsymbol{\nu }}}\}$ are solution of the linear system
  $$\begin{aligned} {\boldsymbol{\alpha }}\big ( a_{{\boldsymbol{\mu }}{\boldsymbol{\nu }}}\big )=\big ( \delta _{{\boldsymbol{\mu }}}^{{\boldsymbol{\nu }}}\big )={\textbf {Id}}_{\vert \mathcal {I}_k\vert \times \vert \mathcal {I}_k\vert } \end{aligned}$$
  with
  $$\alpha _{{\boldsymbol{\mu }},{\boldsymbol{\nu }}}=2\vert K\vert \frac{(\mu _1+\nu _1+1)!(\mu _2+\nu _2+1)(\mu _3+\nu _3+1)!}{(2k+1)!}. $$
  This matrix is positive definite.
- The coefficients $a_{{\boldsymbol{\mu }}{\boldsymbol{\nu }}}$ only depend on ${\boldsymbol{\mu }}+{\boldsymbol{\nu }}$, we write $a_{{\boldsymbol{\mu }}{\boldsymbol{\nu }}}=a_{{\boldsymbol{\mu }}+{\boldsymbol{\nu }}}$.

#### Proof

We need to define the $\varphi _{\boldsymbol{\mu }}$s. Since $\langle \delta _\sigma , \varphi _{\boldsymbol{\mu }}\rangle =0$ for all $\sigma$, we see that

$$\begin{aligned} \varphi _{{\boldsymbol{\mu }}}=\lambda _1\lambda _2\lambda _3\big (\sum _{{\boldsymbol{\nu }}\in \mathcal {I}_k}a_{{\boldsymbol{\mu }}{\boldsymbol{\nu }}}\lambda ^{{\boldsymbol{\nu }}}\big ) \end{aligned}$$

and from the second set of family, that for all ${\boldsymbol{\nu }}'$,

$$\begin{aligned} \sum _{{\boldsymbol{\nu }}\in \mathcal {I}_k}\alpha _{{\boldsymbol{\mu }}{\boldsymbol{\nu }}}\int _K \lambda _1\lambda _2\lambda _3 {\boldsymbol{\lambda }}^{{\boldsymbol{\nu }}}{\boldsymbol{\lambda }}^{{\boldsymbol{\nu }}'}\; \mathrm d\textbf{x}=\delta _{{\boldsymbol{\mu }}}^{{\boldsymbol{\nu }}'}. \end{aligned}$$

We have

$$\int _K \lambda _1\lambda _2\lambda _3 {\boldsymbol{\lambda }}^{{\boldsymbol{\mu }}}{\boldsymbol{\lambda }}^{{\boldsymbol{\nu }}}\; \mathrm d\textbf{x}= 2\vert K\vert \frac{(\mu _1+\nu _1+1)!(\mu _2+\nu _2+1)(\mu _3+\nu _3+1)!}{(2k+1)!} $$

because ${\boldsymbol{\mu }}, {\boldsymbol{\nu }}\in \mathcal {I}_k$ so that $2+\sum _i\mu _i+\sum _i\nu _i+3=2(k-2)+5=2k+1$. For the same reason as above, this matrix is invertible because it is positive definite.

We also see that since $\alpha _{{\boldsymbol{\mu }},{\boldsymbol{\nu }}}$ depends only on ${\boldsymbol{\mu }}+{\boldsymbol{\nu }}$, using for example Cramer’s formula, we get that $a_{{\boldsymbol{\mu }}{\boldsymbol{\nu }}}$ only depends on ${\boldsymbol{\mu }}+{\boldsymbol{\nu }}$. $\square$

For example for $k=3$ (which is the first non trivial example), we have, for the moment $m_{\boldsymbol{\mu }}$ with ${\boldsymbol{\mu }}=(\mu _1,\mu _2,\mu _3)$ where the $\mu _1+\mu _2+\mu _3=1$, all the $\mu _j$s are all equal to 0 except for one which is equal to 1. This leads to

$$\begin{aligned} \varphi _{\boldsymbol{\mu }}=\lambda _1\lambda _2\lambda _3(a_{1}^k\lambda _1+a_{2}^k\lambda _2+a_3^k\lambda _3). \end{aligned}$$

The matrix

$$A=\frac{1}{6!}\begin{pmatrix} 6 &  4&  4 \\ 4& 6& 4 \\ 4& 4& 6\end{pmatrix}$$

is circulant. Since $X{=}(a_1,a_2,a_3)^T= (\tfrac{1800}{7},-\frac{720}{7},-\frac{720}{7})^T$ is the solution of

$$\begin{aligned} A X=\begin{pmatrix} 1\\ 0 \\ 0\end{pmatrix} \end{aligned}$$

the polynomials are

- Moment $$\lambda _1$$: $$\varphi _{(1,0,0)}=\lambda _1\lambda _2\lambda _3(a_1\lambda _1+a_2\lambda _2+a_3\lambda _3)$$.
- Moment $$\lambda _2$$: $$\varphi _{(0,1,0)}=\lambda _1\lambda _2\lambda _3(a_3\lambda _1+a_1\lambda _2+a_2\lambda _3)$$.
- Moment $$\lambda _3$$: $$\varphi _{(0,0,1)}=\lambda _1\lambda _2\lambda _3(a_2\lambda _1+a_3\lambda _2+a_1\lambda _3)$$.

From Riesz theorem, we know that for any linear form $\varphi ^\star$ defined on *V*, there exists an element $\varphi \in V$ such that for all $p\in V$,

$$\begin{aligned} \langle \varphi ^\star , p\rangle =\frac{1}{\vert K\vert } \int _K \varphi (\textbf{x})p(\textbf{x})\; \mathrm d\textbf{x}\end{aligned}$$

because

$$\begin{aligned} (p,q)=\frac{1}{\vert K\vert } \int _K p(\textbf{x})q(\textbf{x})\; \mathrm d\textbf{x}\end{aligned}$$

is a scalar product on *V*.

From its definition, $m_{{\boldsymbol{\mu }}}$ for ${\boldsymbol{\mu }}\in \mathcal {I}_k$ is associated to ${\boldsymbol{\lambda }}^{\boldsymbol{\mu }}$ since

$$\begin{aligned} \langle m_{\boldsymbol{\mu }}, p\rangle =\frac{1}{\vert K\vert }\int _K{\boldsymbol{\lambda }}^{\boldsymbol{\mu }}p\; \mathrm d\textbf{x}. \end{aligned}$$

We are interested in looking at $\psi _\sigma$ such that for all $p\in V$,

$$\begin{aligned} \langle \delta _{\sigma },p\rangle =\frac{1}{\vert K\vert }\int _K \psi _{\sigma } p\; \mathrm d\textbf{x}. \end{aligned}$$

We write

18a $$\begin{aligned} \psi _{\sigma }=\sum \limits _{\sigma '\in \mathcal {G}}\alpha _{\sigma \sigma '}\varphi _{\sigma '}+\sum \limits _{{\boldsymbol{\mu }}'\in \mathcal {I}_k}\alpha _{\sigma {\boldsymbol{\mu }}' }\varphi _{{\boldsymbol{\mu }}'} \end{aligned}$$

and

18b $$\begin{aligned} {\boldsymbol{\lambda }}^{\boldsymbol{\mu }}=\sum \limits _{\sigma '\in \mathcal {G}}\alpha _{{\boldsymbol{\mu }}\sigma '}\varphi _{\sigma '}+\sum \limits _{{\boldsymbol{\mu }}'\in \mathcal {I}_k}\alpha _{{\boldsymbol{\mu }}{\boldsymbol{\mu }}' }\varphi _{{\boldsymbol{\mu }}'} \end{aligned}$$

and get

$$\begin{aligned} \vert K\vert \delta _{\sigma \sigma '}=\sum \limits _{\sigma '\in \mathcal {G}}\alpha _{\sigma \sigma '}\int _K\varphi _{\sigma }\varphi _{\sigma '}\; \mathrm d\textbf{x}+\sum \limits _{{\boldsymbol{\mu }}'\in \mathcal {I}_k}\alpha _{\sigma {\boldsymbol{\mu }}' }\int _K\varphi _{\sigma } \varphi _{{\boldsymbol{\mu }}'}\; \mathrm d\textbf{x}, \end{aligned}$$

$$\begin{aligned} 0=\sum \limits _{\sigma '\in \mathcal {G}}\alpha _{\sigma \sigma '}\int _K\varphi _{{\boldsymbol{\mu }}}\varphi _{\sigma '}\; \mathrm d\textbf{x}+\sum \limits _{{\boldsymbol{\mu }}'\in \mathcal {I}_k}\alpha _{\sigma {\boldsymbol{\mu }}' }\int _K\varphi _{{\boldsymbol{\mu }}} \varphi _{{\boldsymbol{\mu }}'}\; \mathrm d\textbf{x}, \end{aligned}$$

$$\begin{aligned} 0=\sum \limits _{\sigma '\in \mathcal {G}}\alpha _{{\boldsymbol{\mu }}\sigma '}\int _K\varphi _\sigma \varphi _{\sigma '}\; \mathrm d\textbf{x}+\sum \limits _{{\boldsymbol{\mu }}'\in \mathcal {I}_k}\alpha _{{\boldsymbol{\mu }}{\boldsymbol{\mu }}' }\int _K\varphi _\sigma \varphi _{{\boldsymbol{\mu }}'}\; \mathrm d\textbf{x}, \end{aligned}$$

$$\begin{aligned} \vert K\vert \delta _{{\boldsymbol{\mu }}{\boldsymbol{\mu }}'}=\sum \limits _{\sigma '\in \mathcal {G}}\alpha _{{\boldsymbol{\mu }}\sigma '}\int _K\varphi _{\boldsymbol{\mu }}\varphi _{\sigma '}\; \mathrm d\textbf{x}+\sum \limits _{{\boldsymbol{\mu }}'\in \mathcal {I}_k}\alpha _{{\boldsymbol{\mu }}{\boldsymbol{\mu }}' }\int _K\varphi _{{\boldsymbol{\mu }}} \varphi _{{\boldsymbol{\mu }}'}\; \mathrm d\textbf{x}\end{aligned}$$

that is

18c $$\begin{aligned} \begin{aligned} \vert K\vert&\begin{pmatrix} {\textbf {Id}}_{(3k)\times (3k)} &  \textbf{0}_{(3k)\times \vert \mathcal {I}_k\vert }\\ \textbf{0}_{\vert \mathcal {I}_k\vert \times (3k)}& {\textbf {Id}}_{\vert \mathcal {I}_k\vert \times \vert \mathcal {I}_k\vert }\end{pmatrix} = \begin{pmatrix} (\alpha _{\sigma \sigma '})_{(3k)\times (3k)} &  (\alpha _{\sigma {\boldsymbol{\mu }}})_{(3k)\times \vert \mathcal {I}_k\vert }\\ (\alpha _{\sigma \sigma '})_{\vert \mathcal {I}_k\vert \times (3k)}&  (\alpha _{\mu {\boldsymbol{\mu }}' })_{\vert \mathcal {I}_k\vert \times \vert \mathcal {I}_k\vert } \end{pmatrix}\\&\qquad \qquad \times \begin{pmatrix} \big ( \int _K\varphi _{\sigma }\varphi _{\sigma '}\; \mathrm d\textbf{x}\big )_{(3k)\times (3k)}&  \big ( \int _K\varphi _{\sigma }\varphi _{{\boldsymbol{\mu }}'}\; \mathrm d\textbf{x}\big )_{(3k)\times \vert \mathcal {I}_k\vert }\\ \big ( \int _K\varphi _{{\boldsymbol{\mu }}}\varphi _{\sigma }\; \mathrm d\textbf{x}\big )_{\vert \mathcal {I}_k\vert \times (3k)}&  \big ( \int _K\varphi _{{\boldsymbol{\mu }}}\varphi _{{\boldsymbol{\mu }}'}\; \mathrm d\textbf{x}\big )_{\vert \mathcal {I}_k\vert \times \vert \mathcal {I}_k\vert } \end{pmatrix}. \end{aligned} \end{aligned}$$

i.e. the matrix of the coordinates of the basis $\{\psi _{{\boldsymbol{\sigma }}},{ {\boldsymbol{\lambda }}}^{{\boldsymbol{\mu }}} \}$ is the inverse of the mass matrix (up-to a factor $1/\vert K\vert$).

Application to the convection problem with constant advection speed. Then we extend what has been done in one dimension. We start from the problem

$$\begin{aligned} \dfrac{\partial u}{\partial t}+\textbf{a}\cdot \nabla u=0 \end{aligned}$$

in *K*, assuming an initial condition in *V*. At $t=0$, since $\textbf{a}$ is constant, and from the definition of the elements of *V*, $\textbf{a}\cdot \nabla u\in V$, and then we have initially

19 $$\begin{aligned} \begin{aligned} \dfrac{\partial }{\partial t}\langle \delta _\sigma , u\rangle&+\langle \delta _\sigma , \textbf{a}\cdot \nabla u\rangle =0\\ \dfrac{\partial }{\partial t}\langle m_{\boldsymbol{\mu }}, u\rangle&+\langle m_{\boldsymbol{\mu }}, \textbf{a}\cdot \nabla u\rangle =0 \end{aligned} \end{aligned}$$

that we can equivalently write as

20 $$\begin{aligned} \begin{aligned} \dfrac{\partial }{\partial t}\int _K\psi _\sigma u\; \mathrm d\textbf{x}&+\int _K \psi _\sigma \textbf{a}\cdot \nabla u\; \mathrm d\textbf{x}=0\\ \dfrac{\partial }{\partial t}\int _K {\boldsymbol{\lambda }}^\mu u\; \mathrm d\textbf{x}&+\int _K {\boldsymbol{\lambda }}^{\boldsymbol{\mu }}\textbf{a}\cdot \nabla u\; \mathrm d\textbf{x}=0.\\ \end{aligned} \end{aligned}$$

Then using (18), and in particular (18c), we get

21 $$\begin{aligned} M\dfrac{\mathrm d}{\mathrm dt}\begin{pmatrix} \int _K\varphi _\sigma u\; \mathrm d\textbf{x}\\ \int _K\varphi _{\boldsymbol{\mu }}u\; \mathrm d\textbf{x}. \end{pmatrix} + \begin{pmatrix} \int _K \varphi _\sigma \textbf{a}\cdot \nabla u\; \mathrm d\textbf{x}\\ \int _K \varphi _{\boldsymbol{\mu }}\textbf{a}\cdot \nabla u\; \mathrm d\textbf{x}\end{pmatrix}=0 \end{aligned}$$

#### Remark 2.3

If, instead of writing the time continuous version of the problem, we would have had started from a semi-discretisation in time, for example the explicit Euler forward scheme,

$$\begin{aligned} u^{n+1}=u^n+\Delta t \textbf{a}\cdot \nabla u^n, \end{aligned}$$

we would have got

$$\begin{aligned} M\bigg (\begin{pmatrix} \int _K\varphi _\sigma u\; \mathrm d\textbf{x}\\ \int _K\varphi _{\boldsymbol{\mu }}u\; \mathrm d\textbf{x}\end{pmatrix}^{n+1}-\begin{pmatrix} \int _K\varphi _\sigma u\; \mathrm d\textbf{x}\\ \int _K\varphi _{\boldsymbol{\mu }}u\; \mathrm d\textbf{x}\end{pmatrix}^{n}\bigg ) + \Delta t\begin{pmatrix} \int _K \varphi _\sigma \textbf{a}\cdot \nabla u\; \mathrm d\textbf{x}\\ \int _K \varphi _{\boldsymbol{\mu }}\textbf{a}\cdot \nabla u\; \mathrm d\textbf{x}\end{pmatrix}=0. \end{aligned}$$

This is maybe a more rigorous way to proceed.

The method can be reinterpreted as in one dimension. We start from the dG approximation, setting

$$\begin{aligned} u_{\mathrm h}=\sum _{\sigma }\langle \delta _\sigma , u\rangle \varphi _\sigma +\sum _{{\boldsymbol{\mu }}} \langle m_{\boldsymbol{\mu }},u\rangle \phi _{\boldsymbol{\mu }}, \end{aligned}$$

let this evolve in time by

$$\begin{aligned} \texttt{M}\dfrac{\mathrm d}{\mathrm dt}\textbf{U}+\textbf{F}=0 \end{aligned}$$

with

$$\begin{aligned} \textbf{U}=\big (\{\langle \delta _\sigma ,u\rangle \}, \{\langle m_{\boldsymbol{\mu }}, u\rangle \}\big )^T \end{aligned}$$

and $$\textbf{F}=(\{F_\sigma \}, \{F_{\boldsymbol{\mu }}\})^T$$ with

$$\begin{aligned} F_\sigma =-\int _K \nabla \varphi _\sigma \cdot \textbf{f}(u_{\mathrm h})\;\mathrm d\textbf{x}+\oint _{\partial K}\varphi _\sigma \textbf{f}(u_{\mathrm h})\cdot \textbf{n}\; \mathrm d\gamma \end{aligned}$$

and

$$\begin{aligned} F_{\boldsymbol{\mu }}=-\int _K \nabla \varphi _{\boldsymbol{\mu }}\cdot \textbf{f}(u_{\mathrm h})\;\mathrm d\textbf{x}+\oint _{\partial K}\varphi _{\boldsymbol{\mu }}\textbf{f}(u_{\mathrm h})\cdot \textbf{n}\; \mathrm d\gamma \end{aligned}$$

and then we get

$$\begin{aligned} \dfrac{\mathrm dU}{\mathrm dt}+M^{-1} \begin{pmatrix} F_\sigma \\ F_{\boldsymbol{\mu }}\end{pmatrix}=0. \end{aligned}$$

#### Remark 2.4

*(Scheme for quadrangles)* The calculations are the same once we have evaluated the basis functions. They can be evaluated in the same way as for triangles.

#### Remark 2.5

*(Scheme for Polygons)* The extension of [19] to polygons has been made in [14]. The extension of what has been done in this section to polygons is quite natural, and will be the topic of a future publication.

### Interpretation of the PamPa Scheme.

Again, we are dealing with the linear advection equation, with a constant speed. We will introduce the flux, $\textbf{f}(u)=\textbf{a}\, u$. For the sake of simplicity, we reduce ourselves to the case of third order, i.e. DoFs are point values and average, and we discuss only the one dimensional and triangular cases for simplicity. In the triangular case, again $\lambda _1$, $\lambda _2$ and $\lambda _3$ are the barycentric coordinates. The dG scheme in one element writes

$$\begin{aligned} \texttt{M}_K\dfrac{\mathrm dU_K}{\mathrm dt}+F=0 \end{aligned}$$

where $U_K=(\{u_{\sigma }\}_{\sigma \in K}, \bar{u}_K)$ and $F=(\{F_{\sigma }\}_{\sigma \in K}, \bar{F}_K)$ with

$$\begin{array}{l}F_{\sigma }= -\int _{{K}} \nabla \varphi _{\sigma } \cdot \textbf{f}(u_{\textrm{h}})\; \mathrm d\textbf{x}+\int _{\partial K}\varphi _{\sigma }\textbf{f}(u_{\textrm{h}})\cdot \textbf{n}\; \mathrm d\gamma ,\\ ~ \\ \bar{F}_K=-\int _{{K}} \nabla \bar{\varphi } \cdot \textbf{f}(u_{\textrm{h}})\; \mathrm d\textbf{x}+\int _{\partial K}\bar{\varphi }\textbf{f}(u_{\textrm{h}})\cdot \textbf{n}\; \mathrm d\gamma \end{array} $$

The Euler forward is

22a $$\begin{aligned} U_{\vert K}^{n+1}=U_{\vert K}^n-\Delta t M_K^{-1}F_K \end{aligned}$$

We note that

$$\begin{aligned} M_K^{-1}=P_K=\frac{1}{|K|} \mathcal {P} \end{aligned}$$

where the matrix $\mathcal {P}$ does not depend on *K*, because we are using barycentric coordinate: this is as if working in the reference element. From (22a), and using the previous results, we see that the average evolves as

$$\begin{aligned} \bar{u}_K^{n+1}=\bar{u}_K^n-\frac{\Delta t}{\vert K\vert }\int _{\partial K} \textbf{f}(u_{\textrm{h}})\cdot \textbf{n}\; \mathrm d\gamma \end{aligned}$$

and the point values as

$$\begin{aligned} u_{\sigma ,K}^{n+1}=u_\sigma ^n-\frac{\Delta t }{\vert K\vert }\bigg (\mathcal {P}^{-1}F_K\bigg )_\sigma \end{aligned}$$

where, using the previous results, we have

$$\begin{aligned} \bigg (\mathcal {P}^{-1}F_K\bigg )_\sigma =\int _{K}\nabla \varphi _\sigma \cdot \textbf{f}(u_{\textrm{h}})\; \mathrm d\textbf{x}+\int _{\partial K}\varphi _{\sigma }\textbf{f}(u_{\textrm{h}})\cdot \textbf{n}\; \mathrm d\gamma = \textbf{a}\cdot \nabla u_{\textrm{h}}(\sigma ). \end{aligned}$$

We have written $u_{\sigma ,K}^{n+1}$ to emphasis that there are several values of $u_\sigma$ at time $n+1$. At time $t_n$, we have data in *V*, so that we need to project the family $\{u_{\sigma ,K}^{n+1}\}$ onto *V*. One possibility is then: change nothing for the average, and for the points we do:

22b $$\begin{aligned} u_\sigma ^{n+1}= \sum _{K, \sigma \in K}{\omega }_{K,\sigma }u_{\sigma ,K}^{n+1} \end{aligned}$$

with weight ${\omega }_{\sigma ,K}$ that are assumed to be positive and satisfy

$$\begin{aligned} \sum _{K, \sigma \in K}\omega _{K,\sigma }={\textbf {Id}}\end{aligned}$$

to define a projection. This amount to write

$$\begin{aligned} u_\sigma ^{n+{1}}= u_\sigma ^n-\Delta t \sum _{K, \sigma \in K} \frac{\omega _{K,\sigma }}{\vert K\vert }\big (\mathcal {P}^{-1}F_{K}\big )_{\sigma }. \end{aligned}$$

The next question is how to choose the weights $\omega _{K,\sigma }$? Looking back at [10] with this interpretation, where the mesh is $\{x_j\}_{j\in \mathbb {Z}}$ and the elements are $K_{j+1/2}=[x_j, x_{j+1}]$, the choice amounts to be, for $\sigma =x_j$

$$\begin{aligned} \omega _{K_{j+1/2},x_j}=\frac{a^+}{a^+ +(-a)^+}, \quad \omega _{K_{j-1/2},x_j}=\frac{(-a)^+}{a^+ +(-a)^+}, \end{aligned}$$

that is

$$\begin{aligned} \omega _{K_{j+1/2},x_j}=\text {sign}(a), \quad \omega _{K_{j-1/2},x_j}=\text {sign}(-a). \end{aligned}$$

However, other choices are certainly possible. It is easy to see that in 1D, there is an energy inequality when we project with a simple averaging procedure: assume now that the scheme writes

$$\begin{aligned} \textbf{u}^{n+1}_j=\dfrac{ \Delta _{j+1/2}{u_{j, j+1/2}^{n+1}}+\Delta _{j-1/2}u_{j, j-1/2}^{ n+1}}{\Delta _{j+1/2}+\Delta _{j-1/2}} \end{aligned}$$

with $$\Delta _{l+1/2}=x_{l+1}-x_l$$.

This defines an energy diagonal matrix, i.e. a norm, and one can show that

#### Proposition 2.6

We have the following energy inequality

23 $$\begin{aligned} \begin{aligned} \sum _{j}(\Delta _{j+1/2}+\Delta _{j-1/2})(u_j)^2&= \sum _{j}(\Delta _{j+1/2}+\Delta _{j-1/2})\bigg ( \dfrac{ \Delta _{j+1/2}u_{j, j+1/2}+\Delta _{j-1/2}u_{j, j-1/2}}{\Delta _{j+1/2}+\Delta _{j-1/2}}\bigg )^2\\  &\le \sum _{K}\Delta _{j+1/2}\big ((u_{j,j+1/2})^2+(u_{j,j-1/2})^2\big )^2 \end{aligned} \end{aligned}$$

#### Proof

For any $A,B,\alpha ,\beta \in \mathbb {R}$ with $\alpha , \beta \ge 0$ and $\alpha +\beta =1$, we have

$$(\alpha A+\beta B)^2= \alpha ^2 A^2+\beta ^2 B^2 +2\alpha \beta AB\le \alpha ^2 A^2+\beta ^2 B^2+\alpha \beta (A^2+B^2)=\alpha A^2+\beta B^2$$

then setting

$$\begin{aligned} \beta _{j}^+=\dfrac{\Delta _{j+1/2}}{\Delta _{j+1/2}+\Delta _{j-1/2}}, \alpha _{j}^{-}=\dfrac{\Delta _{j-1/2}}{\Delta _{j+1/2}+\Delta _{j-1/2}}, \gamma _{j}=\Delta _{j+1/2}+\Delta _{j-1/2} \end{aligned}$$

$$\begin{aligned} \begin{aligned}\sum _j \gamma _j u_j^2&=\sum _{j}\gamma _j\big (\alpha _{j}^-u_{j,j-1/2} + \beta _{j}^{+}u_{j,j+1/2}\big )^2\le \sum _j \gamma _j (\alpha _{j}^-) (u_{j,j-1/2})^2+(\beta _{j}^{+})(u_{j,j+1/2})^2)\\&=\sum _{K=[x_j,x_{j+1}]} \bigg (\gamma _j\beta _{j}^-(u_{j,j+1/2})^2+\gamma _{j+1}\alpha _{j+1}^-(u_{j+1,j+1/2})^2\bigg ) \end{aligned} \end{aligned}$$

Then,

$$\begin{aligned} \begin{aligned}\sum _j \gamma _j u_j^2&=\sum _{j}\gamma _j\big (\alpha _{j}^-u_{j}^- + \beta _{j}^{+}u_{j}^+\big )^2\le \sum _j \gamma _j (\alpha _{j}^-) (u_{j}^-)^2+(\beta _{j}^{+})(u_{j}^+)^2)\\&=\sum _{K} \bigg (\gamma _j\beta _{j}^-(u_j^+)^2+\gamma _{j+1}\alpha _{j+1}^-(u_{j+1}^+)^2\bigg )\\&\le \sum _K\Delta _{j+1/2}\bigg ( (u_j^+)^2+(u_{j+1}^+)^2\bigg ) \end{aligned} \end{aligned}$$

because

$$\gamma _j\beta _{j}^+=\Delta _{j+1/2} \frac{\Delta _{j+1/2}}{\Delta _{j-1/2}+\Delta _{j+1/2}}\le \Delta _{j+1/2} $$

and

$$ \gamma _j\alpha _{j+1}^-= \Delta _{j+1/2}\dfrac{\Delta _{j+1/2}}{\Delta _{j+3/2}+\Delta _{j+1/2}} \le \Delta _{j+1/2}.$$

which is nothing more than (23) when $\textbf{u}_j^\pm =\textbf{u}_{j, j\pm 1/2}$, etc $\square$

**Fig. 2:**
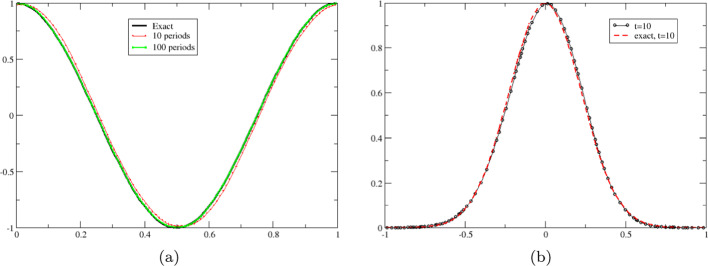
Solution of $u_t+u_x=0$ with periodic boundary conditions on [0, 1] for the initial condition $u_0=\cos (2\pi x)$ after 10 and 100 periods, compared to the exact solution, on regular mesh (fig. a). On fig. (b), the initial solution is $u_0=e^{-10x^2}$ on $[-1,1]$ after 10 rotations on a random mesh.

This strategy, in 1D, is illustrated on Fig. 2 where the problem is that of the convection of $\cos (2\pi x)$ with constant speed. The plot shows on a 100 points grid the result after 10 and 100 periods compared to the exact solution. We see very little dispersion.

In two dimensions, the choice (22b) with weights defining an arithmetic average or an average weight by the area of the elements, as inspired by the 1D case, seems to lead to unstable results, but the choice

24 $$\begin{aligned} \tilde{\omega }_{\sigma ,K}=\bigg (\sum _{K, \sigma \in K} \big (\text {sign}(\nabla \textbf{f}(\textbf{u}_\sigma )\bullet \textbf{n}_\sigma ^K) +\varepsilon {\textbf {Id}}_k\big )\bigg )^{-1}\; \bigg ( \text {sign}\big (\nabla \textbf{f}(\textbf{u}_\sigma )\bullet \textbf{n}_\sigma ^K\big )+\varepsilon {\textbf {Id}}_k\bigg ) \end{aligned}$$

where, for $\textbf{A}=(A,B)$, $A,B\in M_k(\mathbb {R})$ and $\textbf{n}=(n_x,n_y)$, $\textbf{A}\bullet \textbf{n}=An_x+Bn_y$ and $\varepsilon$ is a small positive parameter $\approx 10^{-20}$ to avoid inversion problems^2^. This choice was made in [19], and then in [14] in the case of polygons.

This is illustrated by the following results, where, in $\Omega =[-20,20]$,

$$\begin{aligned} \textbf{u}_0(\textbf{x})=\exp (-\alpha \Vert \textbf{x}-\textbf{x}_0\Vert ^2), \alpha =0.25 \end{aligned}$$

on the problem

25 $$\begin{aligned} \dfrac{\partial \textbf{u}}{\partial t}+\text { div }\textbf{f}(\textbf{u})=0 \end{aligned}$$

with

26a $$\begin{aligned} \textbf{f}(u)=\textbf{a}u, \quad \textbf{a}(=-1,-1), \quad \textbf{x}_0=(15,15) \end{aligned}$$

and final time $T=30$ or

26b $$\begin{aligned} \textbf{f}(\textbf{x}, \textbf{u})=2\pi (y,-x)u, \textbf{x}=(x,y), \quad \textbf{x}_0=(-10,0) \end{aligned}$$

The final time is $T=1$, i.e. one full rotation.

The tables 1 and 2 show the error for the scheme with upwind weights for (25) with the flux (26). The CFL is set to 0.3.

**Table 1:** Errors for the average and point values, triangular mesh, translation problem (25)-(26a) $T=30$.

Average values						
*h*	$L^1$	slope	$L^2$	slope	$L^\infty$	slope
0.4100	$0.4412\;10^{-3}$	-	$0.4412\;10^{-3}$	-	$0.4901\;10^{-1}$	-
0.2975	$0.1958\;10^{-3}$	2.533	$0.1224\;10^{-2}$	2.443	$0.2313\;10^{-1}$	2.341
0.2442	$0.1009\;10^{-3}$	3.361	$0.6420\;10^{-3}$	3.274	$0.1233\;10^{-1}$	3.189
0.2083	$0.5819\;10^{-4}$	3.460	$0.3732\;10^{-3}$	3.409	$0.7148\;10^{-2}$	3.429
0.1533	$0.2073\;10^{-4}$	3.364	$0.1347\;10^{-3}$	3.321	$0.2621\;10^{-2}$	3.270

Point values						
0.4100	$0.2915\;10^{-3}$	-	$0.2915\;10^{-3}$	-	$0.5018\;10^{-1}$	-
0.2975	$0.1295\;10^{-3}$	2.528	$0.1006\;10^{-2}$	2.456	$0.2338\;10^{-1}$	2.381
0.2442	$0.6689\;10^{-4}$	3.351	$0.5262\;10^{-3}$	3.285	$0.1238\;10^{-1}$	3.222
0.2083	$0.3863\;10^{-4}$	3.451	$0.3056\;10^{-3}$	3.416	$0.7208\;10^{-2}$	3.401
0.1533	$0.1379\;10^{-4}$	3.357	$0.1102\;10^{-3}$	3.325	$0.2633\;10^{-2}$	3.284

**Table 2:** Errors for the average and point values, triangular mesh, rotation problem (25)-(26b), $T=1$.

Average values						
*h*	$L^1$	slope	$L^2$	slope	$L^\infty$	slope
0.4100	$0.4575\,10^{-3}$	-	$0.4575\,10^{-3}$	-	$0.7373\,10^{-1}$	-
0.2975	$0.3017\,10^{-3 }$	1.297	$0.1883\,10^{-2}$	2.403	$0.3568\,10^{-1}$	2.262
0.2442	$0.1551\,10^{-3 }$	3.376	$0.9853\,10^{-3}$	3.284	$0.1900\,10^{-1}$	3.197
0.2083	$0.8885\,10^{-4}$	3.500	$0.5702\,10^{-3}$	3.437	$0.1118\,10^{-1}$	3.334
0.1533	$0.3156\,10^{-4}$	3.375	$0.2042\,10^{-3}$	3.349	$0.4074\,10^{-2}$	3.290

Point values						
*h*	$L^1$	slope	$L^2$	slope	$L^\infty$	slope
0.4100	$0.6794\,10^{-3}$	-	$0.6794\,10^{-3}$	-	$0.7538\,10^{-1}$	-
0.2975	$0.2034\,10^{-3}$	3.760	$0.1549\,10^{-2}$	2.436	$0.3596\,10^{-1}$	2.307
0.2442	$0.1041\,10^{-3 }$	3.395	$0.8083\,10^{-3 }$	3.298	$0.1922\,10^{-1}$	3.178
0.2083	$0.5968\,10^{-4 }$	3.498	$0.4671\,10^{-3 }$	3.446	$0.1125\,10^{-1}$	3.363
0.1533	$0.2115\,10^{-4 }$	3.382	$0.2042\,10^{-3}$	2.699	$0.4084\,10^{-2}$	3.305

This shows that the scheme delivers the expected error, even a little bit more. This is likely be a coincidence.

### Generatisation to Non Constant Advection Speeds and Non Linear Problems.

In [19] and [14], the update of the average is done by

$$\begin{aligned} \vert K\vert \dfrac{\mathrm d\bar{u}_K}{\mathrm dt}+\int _{\partial K}\textbf{f}(u)\cdot \textbf{n}\; \mathrm d\gamma =0, \end{aligned}$$

while the update of the point values is done by

$$\begin{aligned} \dfrac{\mathrm d u_{\sigma }}{\mathrm dt}+\sum _{K, \sigma \in K}\omega _{{\boldsymbol{\sigma }}, K} \textbf{J}(u_{\boldsymbol{\sigma }})\nabla u_{\vert K}({\boldsymbol{\sigma }})=0 \end{aligned}$$

where $\textbf{J}$ is the Jacobian of the flux with respect to the conservative variables and $\omega _{{\boldsymbol{\sigma }}, K}$ is obtained by a generalisation of (24), see [14, 19] for details. The main difference with the linear convection problem with constant speed is that $\text {div }\textbf{f}(u)$ is not an element of *V*, so that the equivalence is lost. Nevertheless, one can extend what we have already written in that case, it will be a different scheme that needs to be studied. This interpretation of the PamPa scheme allows in particular to understand how to discretise boundary conditions, see [20] for such a study that is above the present contribution.

## Intrinsic Positivity Properties of PamPa

In all the examples described bellow, as well as in [14], the solution of (1) is approximated by

$$\begin{aligned} \textbf{u}_{\mathrm h}=\sum _{\sigma \in \partial K} \textbf{u}_\sigma \varphi _\sigma +\bar{\textbf{u}}_K \bar{\varphi }_K \end{aligned}$$

where

- $$\bar{\varphi }_K=0$$ on $$\partial K$$, and $$\int _K \bar{\varphi }_K\;\mathrm d\textbf{x}=|K|$$,
- $\varphi _\sigma (\sigma ')=\delta _\sigma ^{\sigma '}$, and for all $\sigma$,
  $$\begin{aligned} \int _K\varphi _\sigma \; \mathrm d\textbf{x}=0 \end{aligned}$$

We will call $\bar{\varphi }$ a bubble function, and in each cases, it admits a maximum that we will denote by $\textbf{x}^\star$. This maximum is in the interior of the polygon.

In this section, we show the following result:

### Proposition 3.1

Let *K* be an element or a polygon. For the 1D third order scheme, the 2D quadratic, cubic, cubic moment and the scheme of [14], we have the following property: There exists $c_0>0$ depending only on *K* with the following property: If $\textbf{u}^n_\sigma \in \mathcal {D}$ and $\textbf{u}(\textbf{x}^\star )\in \mathcal {D}$ then

$$\begin{aligned} \bar{\textbf{u}}^{n+1}_K=\bar{\textbf{u}}^n_K-\frac{\Delta t}{\vert K\vert }\oint _{\partial K}\textbf{f}(\textbf{u}_{\mathrm h})\cdot \textbf{n}\; \mathrm d\gamma \end{aligned}$$

satisfies $$\bar{\textbf{u}}^{n+1}_K\in \mathcal {D}$$ if

$$\begin{aligned} \Delta t\max _{\textbf{x}\in K}\rho (\nabla \textbf{f}(\textbf{u}_{\mathrm h}(\textbf{x})))\le c_0\frac{\vert K\vert }{\vert \partial K\vert }. \end{aligned}$$

In order to establish this property, we proceed in 2 steps. First we show the following lemma

### Lemma 3.2

For the functional approximations mentioned in proposition 3.1, there exists $\textbf{x}^\star \in \text {interior of } K$ such that

$$\begin{aligned} \textbf{u}_{\mathrm h}(\textbf{x}^\star )=\sum _{\sigma \in \partial K} \alpha _\sigma ^K \textbf{u}_\sigma +\omega _K\bar{u}_K \end{aligned}$$

with

$$\begin{aligned} \omega _K>0 \text { and } \alpha _\sigma ^K<0. \end{aligned}$$

In a second step, we use this property, which can be seen as a generalization of Simpson’s formula, to established proposition 3.1.

In general we will have $\textbf{u}(\textbf{x}^\star ), \textbf{u}_\sigma \in \mathcal {D}$ will imply that $\bar{u}_K\in \mathcal {D}$. But the converse is wrong. For example $u=6x(1-x)+7x(3x-2)$ is such that $u(0)=0$, $u(1)=7$ and $\bar{u}=1$, but $u(1/2)=-1/4<0$. This means that when $u(\textbf{x}^\star )\not \in \mathcal {D}$, something else must be done. One possibility is described in [14] where a monolitic convex limiting is used. Another possibility, getting inspiration from dG is to "limit" $\textbf{u}$ in *K* while keeping the average. Such a solution is described in [13], the price to pay is to use numerical flux to update the average value. What we conjecture is that a result of the type described in Lemma 3.2 is always true.

### The 1D Case.

We begin with the 1D case, repeating [13]. The computational domain is covered by a set of non-overlapping cells denoted by $I_{j+\frac{1}{2}}=[x_j,x_{j+1}]$ centered at $x_{j+\frac{1}{2}}$. The solution given in terms of boundary DoFs $u_j^n\approx u(x_j,t^n)$ and internal DoFs $\overline{u}_{j+\frac{1}{2}}\approx \int _{I_{j+\frac{1}{2}}}u(x)\;\mathrm dx/\Delta x_{j+1/2}$ are assumed to be available. For each cell $I_{j+\frac{1}{2}}$, the internal DoF is evolved with

27 $$\begin{aligned} \overline{u}_{j+1/2}^{n+1}=\overline{u}_{j+1/2}^n-\lambda \big (f(u_{j+1})-f(u_j)\big ) \end{aligned}$$

where $\lambda =\frac{\Delta t}{\Delta x_{j+1/2}}$ with $\Delta x$ being the measure of the cell $I_{j+\frac{1}{2}}$ and $\Delta t$ the adaptive time step depends on a certain CFL condition. We consider the third-order of accuracy here and thus can apply the Simpson’s rule to write

$$\begin{aligned} \overline{u}_{j+1/2}^n=\frac{1}{6}\big ( u_j^n+4 u_{j+1/2}^n+u_{j+1}^n\big ), \end{aligned}$$

so that (27) becomes

$$\begin{aligned} \begin{aligned} \overline{u}_{j+1/2}^{n+1}&=\frac{1}{6}\bigg (u_{j+1}^n-6\lambda \big (f(u_{j+1})-{\widehat{f}}(u_{j+1}, u_{j+1/2})\big )\bigg )\\&+\frac{4}{6}\bigg ( {u}_{j+1/2}^n-\frac{6}{4}\lambda \big ({\widehat{f}}(u_{j+1},{u}_{j+1/2})-{\widehat{f}}({u}_{j+1/2}, u_j)\big )\bigg )\\&+\frac{1}{6}\bigg (u_{j}^n-6\lambda \big ({\widehat{f}}({u}_{j+1/2},u_j)-f(u_j)\big )\bigg )\\ \end{aligned} \end{aligned}$$

Then, we look at the intervals: in the cell $$[x_{j-1}, x_j]$$ we have 3 constant cells with value $$u_{j-1}$$, $$ u_{j-1/2}$$, $$u_j$$ and in the cell $$[x_j,x_{j+1]}]$$, we have 3 constant cells with values $$u_j$$, $$u_{j+1/2}$$, $$u_{j+1}$$, so that we can interpret the term $$f(u_j)$$ as the flux $${\widehat{f}}(u_{j},u_j)$$ between the most right subcell of $$[x_{j-1},x_{j}]$$ and the most left subcell of $$[x_j,x_{j+1}]$$. This shows that if $$6\lambda \le c_0$$ the CFL constant for the monotone flux $${\widehat{f}}$$, if $$u_{j+1/2}$$ is in the bounds [*m*, *M*] as well as the other terms, then $$\bar{u}_{j+1/2}^{n+1}\in [m, M]$$. In the one dimensional cubic case, the bubble function is $$6x(1-x)$$ and it reaches its maximum for $$x=\tfrac{1}{2}$$.

The key points are

- The quadrature formula with positive weights,
- The continuity of the approximation at the boundaries of the cell,

### The 2D Case.

If we can find a point such that

$$\begin{aligned} u(\textbf{x}^\star )=\sum _{\text {Lagrange points}}\omega _{\sigma } u(\textbf{x}_\sigma )+\omega _K \bar{u}_K \end{aligned}$$

with $\omega _K>0$ and $\omega _{\sigma }<0$, we can repeat the same argument.

#### Polynomial Case: Quadratic and Cubic Cases of [19].

For quadratic approximation, we have

$$\begin{aligned} u_{\mathrm h}(\textbf{x})=\sum _\sigma u(\textbf{x}_\sigma )\varphi _\sigma (\textbf{x})+\bar{u}_K \bar{\varphi }_K(\textbf{x}) \end{aligned}$$

with

$$\begin{aligned} \bar{\varphi }_K=60\lambda _1\lambda _2\lambda _3 \end{aligned}$$

$$\begin{aligned} \varphi _{\sigma _i}=(2\lambda _i-1)\lambda _i, \quad i=1,2,3 \end{aligned}$$

and

$$\begin{aligned} \varphi _{\sigma _4}=4\lambda _1\lambda _2-\frac{1}{3}\bar{\varphi }_K,\quad \varphi _{\sigma _5}=4\lambda _2\lambda _3-\frac{1}{3}\bar{\varphi }_K,\quad \varphi _{\sigma _6}=4\lambda _3\lambda _1-\frac{1}{3}\bar{\varphi }_K, \end{aligned}$$

and we see that for the centroid $\textbf{x}_K$,

$$\begin{aligned} \bar{\varphi }_K(\textbf{x}_K)=\frac{60}{27}, \quad \varphi _{\sigma _i}=-\frac{1}{9},\quad i=1,2,3; \quad \varphi _{\sigma _i}(\textbf{x}_K)=\frac{4}{9}-\frac{20}{27}=-\frac{8}{27},\quad i=4,5,6 \end{aligned}$$

i.e.

$$\begin{aligned} u(\textbf{x}_K)=\frac{20}{9}\bar{u}_K-\frac{1}{9}\sum _{i=1}^3 u_{\sigma _i}-\frac{8}{27}\sum _{i=4}^6u_{\sigma _i} \end{aligned}$$

from which we get

$$\begin{aligned} \bar{u}_K=\frac{9}{20}u(\textbf{x}_K)+\frac{1}{20}\sum _{i=1}^3 u_{\sigma _i}+\frac{2}{15}\sum _{i=4}^6u_{\sigma _i}. \end{aligned}$$

For cubic approximation, we have

$$\begin{aligned} \varphi _{\sigma _i}=\frac{1}{2}\lambda _i(3\lambda _i-1)(3\lambda _i-2)-\frac{60}{30}\lambda _1\lambda _2\lambda _3,\quad i=1,2,3 \end{aligned}$$

and

$$\begin{aligned} \begin{aligned} \varphi _{\sigma _4}=\frac{9}{2}\lambda _1\lambda _2(3\lambda _1-1)-\frac{9}{2}\lambda _1\lambda _2\lambda _3, \quad \varphi _{\sigma _5}=\frac{9}{2}\lambda _1\lambda _2(3\lambda _2-1)-\frac{9}{2}\lambda _1\lambda _2\lambda _3,\\ \varphi _{\sigma _6}=\frac{9}{2}\lambda _2\lambda _3(3\lambda _2-1)-\frac{9}{2}\lambda _1\lambda _2\lambda _3, \quad \varphi _{\sigma _7}=\frac{9}{2}\lambda _2\lambda _3(3\lambda _3-1)-\frac{9}{2}\lambda _1\lambda _2\lambda _3,\\ \varphi _{\sigma _8}=\frac{9}{2}\lambda _3\lambda _1(3\lambda _3-1)-\frac{9}{2}\lambda _1\lambda _2\lambda _3, \quad \varphi _{\sigma _9}=\frac{9}{2}\lambda _3\lambda _1(3\lambda _1-1)-\frac{9}{2}\lambda _1\lambda _2\lambda _3, \end{aligned} \end{aligned}$$

and we see that

$$\begin{aligned} u(\textbf{x}_K)=-\sum _{j=1}^3 \frac{2}{27}u(\sigma _i)-\sum _{i=4}^9\frac{1}{6}u(\sigma _i)+\frac{60}{27}\bar{u}_K. \end{aligned}$$

From this we get

$$\bar{u}_K=\frac{9}{20}u(\textbf{x}_K)+\frac{1}{30}\sum _{j=1}^3 u(\sigma _i)+\frac{9}{120} \sum _{i=4}^9u(\sigma _i).$$

We note that

$$\begin{aligned} \frac{9}{20}+\frac{3}{20}+\frac{6}{15}=1 \end{aligned}$$

and

$$\begin{aligned} \frac{9}{20}+\frac{3}{30}+\frac{54}{120}=1. \end{aligned}$$

#### Polynomial Case: Cubic Moment.

We write

$$\begin{aligned} u_{\mathrm h}(\textbf{x})=\sum _{{\boldsymbol{\sigma }}\in \partial K}u_{\boldsymbol{\sigma }}\varphi _{\boldsymbol{\sigma }}(\textbf{x})+\sum _{{\boldsymbol{\mu }}}m_{\boldsymbol{\mu }}(u)\bar{\varphi }_{\boldsymbol{\mu }}(\textbf{x}) \end{aligned}$$

and we evaluate this at the centroid. Because the coefficients defining the $\bar{\varphi }_{\boldsymbol{\mu }}$ are obtained by cyclic permutation, from lemma 2.2, we see that

$$\begin{aligned} \varphi _{\boldsymbol{\mu }}(\frac{1}{3},\frac{1}{3}, \frac{1}{3})=\frac{1}{3^P}\sum _{{\boldsymbol{\nu }}} a_{{\boldsymbol{\mu }}+{\boldsymbol{\nu }}} \end{aligned}$$

where *P* only depends on the degree ($P=3+(k-2)$). This relation shows that

$$\begin{aligned} \omega _K=\varphi _{\boldsymbol{\mu }}(\frac{1}{3},\frac{1}{3}, \frac{1}{3}) \end{aligned}$$

does not depend on ${\boldsymbol{\mu }}$ and, because the sum of the moments is the average, we obtain

$$\begin{aligned} u_{\mathrm h}\left( \frac{1}{3},\frac{1}{3}, \frac{1}{3}\right) =\sum _{\boldsymbol{\sigma }}u_{\boldsymbol{\sigma }}\varphi _{\boldsymbol{\sigma }}\left( \frac{1}{3},\frac{1}{3}, \frac{1}{3}\right) +\omega _K \overline{\textbf{u}}_K \end{aligned}$$

The only thing to check is if $\omega _K>0$ and

$$\begin{aligned} \varphi _{\boldsymbol{\sigma }}\left( \frac{1}{3},\frac{1}{3}, \frac{1}{3} \right) <0. \end{aligned}$$

In the cubic case $(k=3)$, $P=4$ and

$$\begin{aligned} \omega _K=\frac{1}{3^4}\big ( \frac{1800-720-720}{7}\big )=\frac{360}{7\times 3^4}=\frac{360}{567}>0 \end{aligned}$$

and $\varphi _{{\boldsymbol{\sigma }}}(\frac{1}{3},\frac{1}{3}, \frac{1}{3})=P_{\boldsymbol{\sigma }}(\frac{1}{3},\frac{1}{3}, \frac{1}{3})-\sum _{\boldsymbol{\mu }}\frac{m_{\boldsymbol{\mu }}(P_{\boldsymbol{\sigma }})}{m_{\boldsymbol{\mu }}(\varphi _{\boldsymbol{\mu }})}\varphi _{\boldsymbol{\mu }}(\frac{1}{3},\frac{1}{3},\frac{1}{3})$. The Gauss–Lobatto points in [0, 1] are $\{\alpha _0=0,\alpha _1=\tfrac{\sqrt{5}-1}{2\sqrt{5}}, \alpha _2=\tfrac{\sqrt{5}+1}{2\sqrt{5}}, \alpha _3=1\}$. The Lagrange polynomials are (the interpolation points are given by their barycentric coordinates)

- for (1, 0, 0),
  $$\begin{aligned} P_0=\dfrac{\lambda _1(\lambda _1-\alpha _1)(\lambda _1-\alpha _2)}{(1-\alpha _1)(1-\alpha _2)} \end{aligned}$$
  and its value at the centroid is
  $$\begin{aligned} -\frac{1}{27}<0. \end{aligned}$$
- For $(\alpha _2,\alpha _1,0)$, it is
  $$\begin{aligned} P_1=\frac{\lambda _1\lambda _2(\lambda _1-\alpha _1)}{\alpha _2\alpha _1(\alpha _2-\alpha _1)} \end{aligned}$$
  and its value at the centroid is
  $$\begin{aligned} \frac{5}{18}-\frac{5\sqrt{5}}{54}>0 \end{aligned}$$
- for $(\alpha _1,\alpha _2,0)$ it is
  $$\begin{aligned} P_2=\frac{\lambda _1\lambda _2(\lambda _1-\alpha _2)}{\alpha _1\alpha _2(\alpha _1-\alpha _2)} \end{aligned}$$
  and its value at the centroid is
  $$\begin{aligned} \frac{5}{18} + \frac{5\sqrt{5}}{54}>0 \end{aligned}$$
- for (0, 1, 0),
  $$\begin{aligned} P_3=\dfrac{\lambda _2(\lambda _2-\alpha _1)(\lambda _2-\alpha _2)}{(1-\alpha _1)(1-\alpha _2)} \end{aligned}$$
  and its value at the centroid is
  $$\begin{aligned} -\frac{1}{27}<0. \end{aligned}$$

Next, we know the value $\varphi _{\boldsymbol{\mu }}(\frac{1}{3},\frac{1}{3},\frac{1}{3})=\frac{360}{567}$ from lemma 2.2 and the form of $\varphi _{{\boldsymbol{\mu }}}$. Moreover, we can compute $m_{\boldsymbol{\mu }}(\varphi _{\boldsymbol{\mu }})$ and $m_{\boldsymbol{\mu }}(P_{\boldsymbol{\sigma }})$. Finally, $\varphi _{\boldsymbol{\sigma }}(\frac{1}{3},\frac{1}{3},\frac{1}{3})<0$ is verified. So again we have the positivity.

#### Approximation Using Virtual Finite Element (VEM) Approximation

In [14], we have extended the method developed in [19]. The computational domain is covered by a family of non overlapping polygon denoted by *K*, $\Omega =\cup K$. In each polygon, the solution is approximated by an element of

$$\begin{aligned} V_k(K)=\{ v\text { such that } v_{\mid \partial K}\in \mathbb {P}^k(\partial K) \text { and }\Delta v\in \mathbb {P}^{k-2}(K)\}, \end{aligned}$$

where $k\ge 2$. In $\Omega$, the solution will be approximated in

$$\begin{aligned} V_k(\Omega )=\bigg (\bigoplus _K V_k(K)\bigg )\cap C^0(\Omega ). \end{aligned}$$

This kind of approximation was introduced in [18, 21, 22] in a variational framework. This is an extension of the classical finite element techniques, where the “elements” are no longer simplex but general polygons with very mild assumptions on the polygons (essentially that they are star shaped with respect to one point). Hence there is no longer a reference element, so that basis functions must be designed for each polygons. This is theoretically possible, at least analytically, but very cumbersome. Hence the idea behind VEM is to avoid to explicitly use basis functions. We sketch the framework, some more details are given in the appendix, the interested reader is suggested to study [18] for a review. The degrees of freedom are the Gauss-Lobato points on each edge of *K* and the moments

$$\begin{aligned} m_{\boldsymbol{\mu }}(u)=\frac{1}{\vert K\vert }\int _K\bigg (\frac{\textbf{x}-\textbf{x}^\star }{h_K}\bigg )^{\boldsymbol{\mu }}u(\textbf{x})\; \mathrm d\textbf{x}, \qquad \vert {\boldsymbol{\mu }}\vert \le k-2 \end{aligned}$$

where we have introduced the following notations:

- $\textbf{x}^\star$ is a point toward which *K* is star-shaped,
- ${\boldsymbol{\mu }}=(\mu _1, \ldots , \mu _d)$ is a multi-index, $\vert {\boldsymbol{\mu }}\vert =\sum _{i=1}^d\mu _i$.
- If $$\textbf{y}=(y_1, \ldots , y_d)$$,
  $$\begin{aligned} y^{\boldsymbol{\mu }}=\Pi _{i=1}^d y_i^{\mu _i}. \end{aligned}$$
- $\sigma$ is any of the Gauss-Lobatto points.
- In the following $\pi$ is the $L^2$ projector defined on $V_k(K)$ onto $\mathbb {P}^k(K)$, see [21, 22]. It is computable solely with the given DoFs, and there is no need to know the basis functions.
- We use the following notations for the “basis” functions: for each DoF on the boundary, we call $\varphi _\sigma$ the element of $V_k(K)$ such that
  $$\begin{aligned} \varphi _\sigma (\sigma ')=\delta _\sigma ^{\sigma '}, m_{\boldsymbol{\mu }}(\varphi _\sigma )=0, \quad \forall {\boldsymbol{\mu }}, \vert {\boldsymbol{\mu }}\vert \le k-2 \end{aligned}$$
  and by $\varphi _{\boldsymbol{\mu }}$ the element of $V_k(K)$ such that
  $$\forall \sigma , \varphi _{\boldsymbol{\mu }}(\sigma )=0\text { and } m_{{\boldsymbol{\mu }}'}(\varphi _{\boldsymbol{\mu }})=\delta _{{\boldsymbol{\mu }}}^{{\boldsymbol{\mu }}'}.$$

The question we want to address is that of the existence of generalised Simpson rules for polygons which are not assumed to be convex but assumed to be star shaped with respect to one point in *K*. This case is a bit more involved, and we consider the quadratic case only. Again we have on the polygon *K*

$$\begin{aligned} u_{\textrm{h}}(\textbf{x})=\sum _{\sigma \in \partial K}u(\textbf{x}_\sigma )\varphi _\sigma (\textbf{x}_\sigma )+\bar{u}_K\bar{\varphi }_K(\textbf{x}) \end{aligned}$$

where the “basis” functions $\varphi$ are such that:

- $$\Delta \varphi _\sigma =\alpha _\sigma \in \mathbb {R}$$, $$\Delta \bar{\varphi }_K=\alpha _K\in \mathbb {R}$$,
- $$\bar{\varphi }_K=0$$ on $$\partial K$$ and $$\int _K\bar{\varphi }_K\;\mathrm d\textbf{x}=\vert K\vert $$,
- $$\varphi _\sigma (\sigma ')=\delta _\sigma ^{\sigma '}$$, $$\varphi _\sigma \in \mathbb {P}^2(\partial K)$$ and $$\int _K\varphi _\sigma \;\mathrm d\textbf{x}=0$$.

The idea is to find a point $\textbf{x}^\star \in \overset{\circ }{\Omega }$ such that

$$\begin{aligned} u(\textbf{x}^\star )=\sum _{\sigma \in \partial \Omega } u_\sigma \varphi _\sigma (\textbf{x}^\star )+\bar{u}\bar{\varphi }_K(\textbf{x}^\star ) \end{aligned}$$

where

$$\begin{aligned} \bar{\varphi }_K(\textbf{x}^\star )>0\text { and } \bar{\varphi }_\sigma (\textbf{x}^\star )<0 \quad \forall \sigma \in \partial K. \end{aligned}$$

It is easy to show that $\bar{\varphi }_K\ge 0$ on *K*. First $\alpha _K<0$ because

$$\begin{aligned} \alpha _K\vert K\vert =\int _K\bar{\varphi }_K\Delta \bar{\varphi }_K\;\mathrm d\textbf{x}=-\int _K \nabla \bar{\varphi }_K^2\;\mathrm d\textbf{x}+\int _{\partial K}\bar{\varphi }_K\nabla \bar{\varphi }_K\cdot \textbf{n}\;\mathrm d\gamma =-\int _K \nabla \bar{\varphi }_K^2\;\mathrm d\textbf{x}<0 \end{aligned}$$

and then $\Delta \bar{\varphi }_K=\alpha _K<0$, so that the maximum principle shows that $\varphi \ge 0$ on *K*. If $\textbf{x}$ is in the interior of *K*, the same maximum principle (more precisely the mean value theorem) shows that $\bar{\varphi }_K(\textbf{x})>0$.

We call $\textbf{x}^\star$ a point for which $\max \limits _{\textbf{x}\in \Omega }\bar{\varphi }_K(\textbf{x})=\bar{\varphi }_K(\textbf{x}^\star )$. We have $\textbf{x}^\star \in \overset{\circ }{\Omega }$ and $\bar{\varphi }_K(\textbf{x}^\star )>1$ because, since

$$\begin{aligned} \vert K\vert \; \bar{\varphi }_K(\textbf{x}^\star )\ge \int _K\bar{\varphi }_K(\textbf{x})\; \mathrm d\textbf{x}=\vert K\vert , \end{aligned}$$

we see that $\bar{\varphi }_K(\textbf{x}^\star )\ge 1$ and if $\bar{\varphi }_K(\textbf{x}^\star )= 1$, we would have

$$\begin{aligned} 0=\int _K \bar{\varphi }_K(\textbf{x})\; \mathrm d\textbf{x}-\vert K\vert =\int _K\big ( \bar{\varphi }_K(\textbf{x})-1\big )\; \mathrm d\textbf{x}\end{aligned}$$

with $\bar{\varphi }_K(\textbf{x})-1\le 0$, so $\bar{\varphi }_K(\textbf{x})-1=0$ and this is not possible.

Next we show that $\alpha _\sigma >0$. Let $\textbf{y}_\sigma$ a point where $\varphi _\sigma$ reaches its minimum. It must be such that $\varphi _\sigma (\textbf{y}_\sigma )<0$ because

$$\begin{aligned} \int _K\varphi _\sigma (\textbf{x})\; \mathrm d\textbf{x}=0. \end{aligned}$$

We have two cases to look at:

- If $\sigma$ is a midpoint, then $\varphi _\sigma \ge 0$ on the boundary. If $\alpha _\sigma \le 0$, then from the maximum principle, $\varphi _\sigma \ge 0$ on $\Omega$ and then we cannot have
  $$\begin{aligned} \int _K\varphi _\sigma (\textbf{x})\; \mathrm d\textbf{x}=0. \end{aligned}$$
  So $$\alpha _\sigma >0$$.
- If $\sigma$ is a vertex: Assume that $\alpha _\sigma <0$. If $\Delta \varphi _\sigma =\alpha _\sigma <0$, then we take $u=\varphi _\sigma +\theta \bar{\varphi }_K$ with $\theta >0$. If the minimum of $\varphi _\sigma$ is reached on the boundary, call it $\textbf{y}^\star$: $\min _\Omega \varphi _\sigma =\min _{\partial \Omega }\varphi _\sigma =\varphi _\sigma (\textbf{y}^\star )$. We have, because $\bar{\varphi }_K\ge 0$, $u\ge \varphi _\sigma$, so $\min _{\Omega } u\ge \min _\Omega \varphi _\sigma$. If the minimum of *u* is reached on $\partial \Omega$
  $$\begin{aligned} \begin{aligned} \varphi _\sigma (\textbf{y}^\star )=\min _\Omega \varphi _\sigma&=\min _{\partial \Omega } \varphi _\sigma =\min _{\partial \Omega }(\varphi _\sigma +\theta \bar{\varphi }_K)\ge \min _\Omega (\varphi _\sigma +\theta \bar{\varphi }_K)\\  &\qquad = \varphi _\sigma (\textbf{y}^\star _\theta )+\theta \bar{\varphi }_K(\textbf{y}^\star _\theta ) >\varphi _\sigma (\textbf{y}^\star _\theta ), \end{aligned} \end{aligned}$$
  so this is absurd. Hence, we must have $\textbf{y}^\star \not \in \partial \Omega$, so since it is a strict minimum, $\Delta \varphi _\sigma (\textbf{y}^\star )=\alpha _\sigma >0$.

We want to show that $\varphi _\sigma (\textbf{x}^\star )<0$.

We take $\varepsilon >0$ and consider $\bar{\varphi }_\varepsilon =\log (\bar{\varphi }_K+\varepsilon )$. It is well defined, and we have

$$\begin{aligned} \nabla \bar{\varphi }_\varepsilon =\frac{\nabla \bar{\varphi }_K}{\bar{\varphi }_K+\varepsilon }, \nabla ^2\bar{\varphi }_\varepsilon =-\frac{\nabla \bar{\varphi }_K\otimes \nabla \bar{\varphi }_K}{(\bar{\varphi }_K+\varepsilon )^2}+\frac{\nabla ^2\bar{\varphi }_K}{\bar{\varphi }_K+\varepsilon }, \end{aligned}$$

so that

$$\begin{aligned} \Delta \bar{\varphi }_\varepsilon =-\frac{\Vert \nabla \bar{\varphi }_K\Vert ^2}{(\bar{\varphi }_K+\varepsilon )^2}+\frac{\Delta \bar{\varphi }_K}{\bar{\varphi }_K+\varepsilon }\le 0. \end{aligned}$$

Then we consider $u=\varphi _\sigma +\theta _\varepsilon \bar{\varphi }_\varepsilon$ for $\theta _\varepsilon \ge 0$. It is clear that for $\varepsilon$ small enough, $u\le 0$ on $\partial \Omega$ if $1+\theta _\varepsilon \log \varepsilon \le 0$ because $\varphi _\sigma \le \max \limits _{\textbf{x}\in \Omega }\varphi _\sigma =1$. So we look for $\epsilon _0$ such that if $\epsilon \le \epsilon _0$,

$$\begin{aligned} \Delta \varphi _\sigma +\theta _\varepsilon \Delta \bar{\varphi }_\varepsilon \le 0 \end{aligned}$$

We have

$$ \Delta \varphi _\sigma +\theta _\varepsilon \Delta \bar{\varphi }_\varepsilon =\underbrace{\alpha _\sigma }_{\ge 0}-\theta _\varepsilon \bigg (\underbrace{\frac{\Vert \nabla \bar{\varphi }_K\Vert ^2}{(\bar{\varphi }_K+\varepsilon )^2}+\frac{\vert \alpha _P\vert }{\bar{\varphi }_K+\varepsilon }}_{\ge 0}\bigg )\le 0$$

if

$$\begin{aligned} \theta _\varepsilon \bigg ( \frac{\Vert \nabla \bar{\varphi }_K\Vert ^2}{(\bar{\varphi }_K+\varepsilon )^2}+\frac{\vert \alpha _P\vert }{\bar{\varphi }_K+\varepsilon }\bigg )\ge {\alpha _\sigma } \end{aligned}$$

so we need

$$\begin{aligned} \theta _\varepsilon \bigg ( \frac{\min _\Omega \Vert \nabla \bar{\varphi }_K\Vert ^2}{(\bar{\varphi }_K(\textbf{x}^\star )+\varepsilon )^2}+\frac{\vert \alpha _P\vert }{\bar{\varphi }_K(\textbf{x}^\star )+\varepsilon }\bigg )\ge {\alpha _\sigma } \end{aligned}$$

Together with the condition on the boundary, we need: so we need

$$ \theta _\varepsilon \bigg ( \dfrac{\min _\Omega \Vert \nabla \bar{\varphi }_K\Vert ^2}{(\bar{\varphi }_K(\textbf{x}^\star )+\varepsilon )^2}+\dfrac{\vert \alpha _P\vert }{\bar{\varphi }_K(\textbf{x}^\star )+\varepsilon }\bigg )\ge {\alpha _\sigma } \text { and } \theta _\varepsilon \log \varepsilon \le -1 $$

i.e

28 $$\begin{aligned} \theta _\varepsilon \bigg ( \dfrac{\min _\Omega \Vert \nabla \bar{\varphi }_K\Vert ^2}{(\bar{\varphi }_K(\textbf{x}^\star )+\varepsilon )^2}+\dfrac{\vert \alpha _P\vert }{\bar{\varphi }_K(\textbf{x}^\star )+\varepsilon }\bigg )\ge {\alpha _\sigma } \text { and } \theta _\varepsilon \log \big (\frac{1}{\varepsilon }\big )\ge 1 \end{aligned}$$

Last we want that

$$\begin{aligned} \varphi _\sigma (\textbf{x}^\star )\le -\theta _\varepsilon \log \big (\bar{\varphi }_K(\textbf{x}^\star )+\varepsilon ) \end{aligned}$$

with

29 $$\begin{aligned} \bar{\varphi }_K(\textbf{x}^\star )+\varepsilon >1 \end{aligned}$$

so with $\varepsilon$ small enough. So we first choose $\varepsilon$ to meet (29) and then we choose $\theta >0$ so that (28) is met. This shows that $\varphi _\sigma (\textbf{x}^\star )<0$.

All in all, we have

$$\begin{aligned} u(\textbf{x}^\star )=\omega _K\bar{u}_K+\sum _{\sigma \in \partial K}\omega _i u_{\sigma _i}, \end{aligned}$$

so that

$$\begin{aligned} \bar{u}_K=\frac{1}{\omega _K}u(\textbf{x}^\star )-\sum _{\sigma \in \partial K}\frac{\omega _i}{\omega _K} u_{\sigma _i}. \end{aligned}$$

Since (take $$u\equiv 1$$)

$$\begin{aligned} 1=\omega _K+\sum _{\sigma \in \partial K}\omega _i, \end{aligned}$$

we see that

$$\begin{aligned} \frac{1}{\omega _K}-\sum _{\sigma \in \partial K}\frac{\omega _i}{\omega _K} =\frac{1}{\omega _K}\big (1-\sum _{\sigma \in \partial K}\omega _i\big )=\frac{\omega _K}{\omega _K}=1. \end{aligned}$$

### Bound Preserving Property

We assume that (28) is such that if $u_0(\textbf{x})\in \mathcal {D}$ for all $\textbf{x}\in \mathbb {R}^d$ (or almost everywhere), then $u(\textbf{x},t)\in \mathcal {D}$ for all $\textbf{x}\in \mathbb {R}^d,t>0$.

In what follows, we assume

$$\begin{aligned} \bar{u}_K=\alpha _K u(\textbf{x}_K)+\sum _{i=1}^N \alpha _i u(\sigma _i) \end{aligned}$$

with $\alpha _K, \alpha _i>0$. We note that

$$\begin{aligned} \alpha _K+\sum _{i=1}^N \alpha _i=1 , \end{aligned}$$

We write ($$\lambda =\tfrac{\Delta t}{\vert K\vert }$$)

$$\begin{aligned} \begin{aligned} \bar{u}_K^{n+1}&=\bar{u}_K^n-\lambda \sum _{i=1}^N \textbf{f}(u_{\sigma _i})\cdot \textbf{n}_i\\&=\alpha _K u(\textbf{x}_K)+\sum _{i=1}^N \alpha _i u(\sigma _i)-\lambda \sum _{i=1}^N \textbf{f}(u_{\sigma _i})\cdot \textbf{n}_i\\&=\alpha _K\bigg ( u(\textbf{x}_K)-\frac{\lambda }{\alpha _K} \sum _{i=1}^N \hat{\textbf{f}}_{\textbf{n}_i}\big (u_{\sigma _i}, u(\textbf{x}_K)\big )\bigg )\\&\qquad +\sum _{i=1}^N \alpha _i\bigg ( u(\sigma _i)-\frac{\lambda }{\alpha _i} \bigg [ \hat{\textbf{f}}_{\textbf{n}_i}(u_{\sigma _i},u_{\sigma _i})\cdot \textbf{n}_i-\hat{\textbf{f}}_{\textbf{n}_i}(u_{\sigma _i}, u(\textbf{x}_K))\bigg ]\bigg ) \end{aligned} \end{aligned}$$

where $\hat{\textbf{f}}_\textbf{n}(\uparrow ,\downarrow )$. Hence, if

$$\begin{aligned} \lambda \min \big (\alpha _K, \min \limits _i\alpha _i\big )\big )\le \lambda _0 \end{aligned}$$

where $\lambda _0$ is the maximum stability parameter for $\hat{\textbf{f}}_\textbf{n}$, we get a convex decomposition. This shows that under this condition, if $\hat{\textbf{f}}_\textbf{n}$ is invariant domain preserving, if $\{u^n_\sigma \in \mathcal {D}\}$ and $\bar{u}^n_K\in \mathcal {D}$ then $\bar{u}_K^{n+1}\in \mathcal {D}$.

We note that there is no need to define any control volume, this is a purely algebraic property.

### Numerical Evidence

**Fig. 3:**
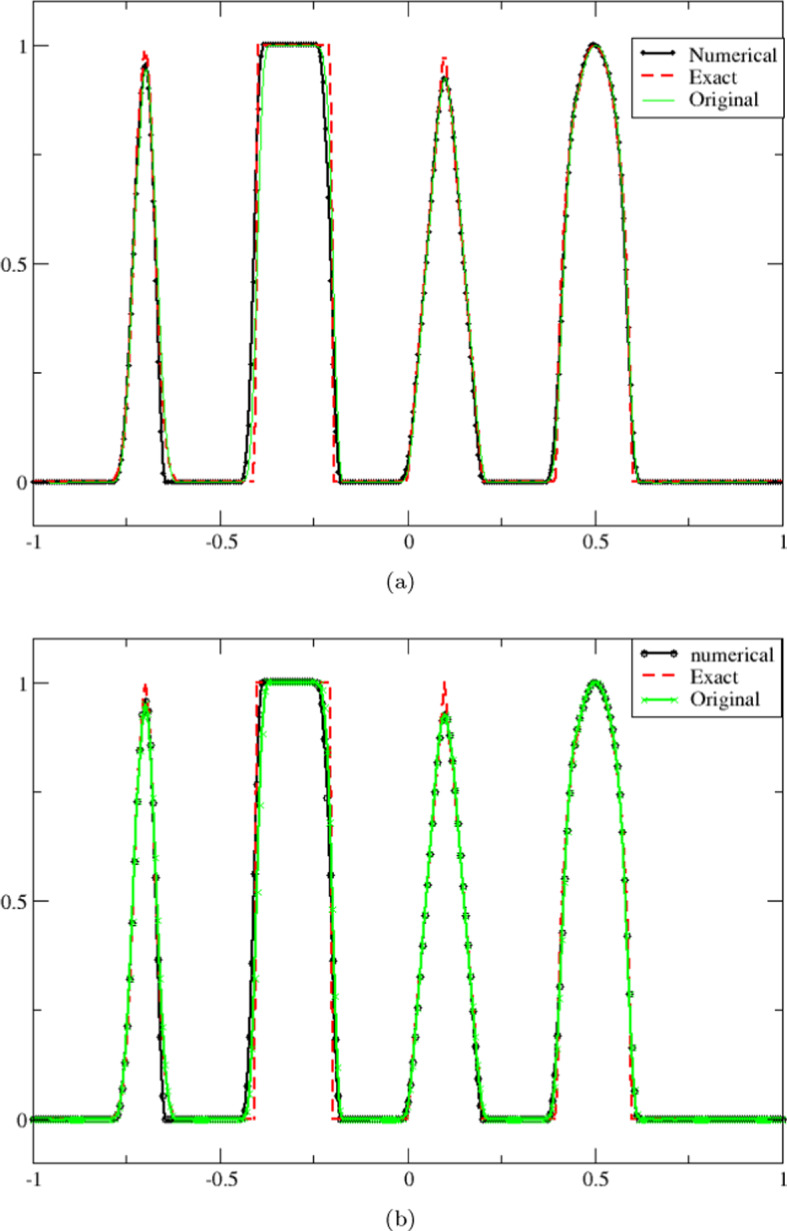
Jiang and Shu’s problem. (a): average values, (b): point values. Original: scheme of [14], Numerical: the BP strategy is applied only on the point values.

In order to illustrate this property, we have used the scheme of [14] on

$$\begin{aligned} \dfrac{\partial u}{\partial t}+\dfrac{\partial u}{\partial x}=0 \end{aligned}$$

on Jiang-Shu problem with periodic boundary conditions, 300 mesh points, until $T=2$ with $CFL=0.15<\tfrac{1}{6}$. The results are displayed in Fig. 3. In the original scheme of [14], the bound preserving strategy is applied both on the point values and the average values. We have tested the results above where we apply the BP strategy only on the point values.

We also consider the compressible Euler equations and show the result of the simulation Configuration 3 of Lax & Liu in [23], the initial condition is

$$ (\rho , u,v,p)=\left\{ \begin{array}{ll} (\rho _1,u_1,v_1,p_1)=(1.5, 0, 0, 1.5)&  \text { if } x\ge 1\text { and } y\ge 1,\\ (\rho _2,u_2,v_2,p_2)=(0.5323, 1.206, 0, 0.3) &  \text { if } x\le 1 \text { and } y\ge 1,\\ (\rho _3,u_3,v_3,p_3)=(0.138, 1.206, 1.206, 0.029)& \text { if } x\le 1\text { and }y\le 1,\\ (\rho _4,u_4,v_4,p_4)=(0.5323, 0, 1.206, 0.3) & \text { if } x\le 1\text { and } y\le 1. \end{array}\right. $$

**Fig. 4:**
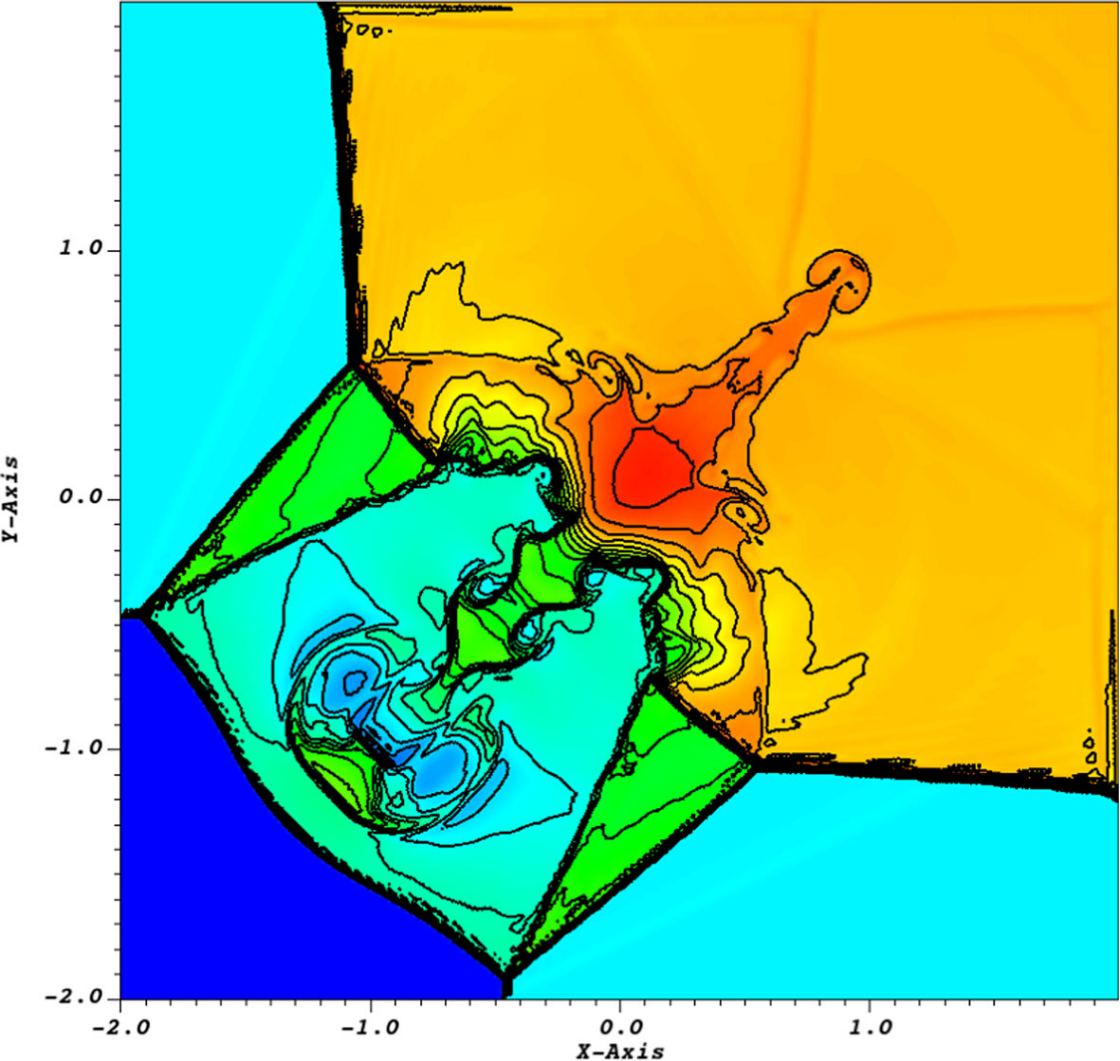
Solution of Lax-Liu case # 3.

Here, the four states are separated by shocks. The domain is $[-2,2]^2$. The solution at $t_f=3$ is displayed in Fig. 4. The mesh is $100\times 100$ cells, i.e. with 120312 DoFs. The scheme is that of [14] where we have applied the bound preserving procedure on the point values only. The CFL is set to 0.1. The spatial approximation uses the Virtual Finite Element formulation.

This is in perfect agreement with proposition 3.1.

## Comment on the Connection to Summation-by-Parts

After our re-interpretation of the PamPa scheme in terms of dG, we are interested on the underlying structures/properties of the PamPa scheme even if we have shown that the scheme is energy stable. One key feature of numerical methods is the summation-by-parts (SBP) property. These schemes mimic integration-by-parts discretely and give a general framework to construct energy-stable schemes [24–26]. Several extensions exists of SBP operators, in addition, in [27], Mattesson introduced upwind SBP operators which have also been applied inside a dG framework together with flux vector splitting more recently in [28]. The key ingredients of SBP or upwind SBP are the following:

- The derivative matrix *D* which approximates the first derivative of a function and has to be exact up to a certain degree *p* of the underlying function space, normally polynomial approximation are used however it is not restricted to it, cf. [29]. In terms of upwind SBP operators, we have two operators $D_+$ and $D_-$ which have to be exact up to a certain degree *p* of the polynomial vector space. They are constructed that $D=(D_++D_-)/2$ holds.
- In classical SBP and upwind SBP framework, a mass/norm matrix *M* which mimics the $L^2$ scalar product and often is set be a diagonal matrix where the entries on the diagonal are the quadrature weights. The exactness of the quadrature has to be at least $2p-1$ for the construction process [30] if a polynomial approximation space is considered.
- The almost skew-symmetric matrix Q:=MD which fulfills than the SBP property meaning
  30 $$\begin{aligned} Q^T+Q=B= {{\,\textrm{diag}\,}}{(-1,0,\dots ,0, 1)} \end{aligned}$$
  for inflow-outflow boundary conditions. For periodic boundary conditions, we have instead
  31 $$\begin{aligned} Q^T+Q=0. \end{aligned}$$
  In terms of the upwind SBP operators, we have $Q_+$ and $Q_-$ such that $Q_++Q^T_-=0$ and $Q_++Q_+^T = S$, where *S* is a dissipation matrix. *S* is symmetric and negative semi-definite. In the upwind case, we have therefore the following conditions:
  32 $$\begin{aligned} \begin{aligned} D_+= M^{-1}(Q_{+} + B/2)&\text { and } D_-= M^{-1}(Q_- + B/2),\\ MD_+ + D^{T}_-M^T=B&\text { and } D_+= D_{-} +M^{-1}S. \end{aligned} \end{aligned}$$

Coming back to the PamPa (9) approach, we can directly define the almost skew-symmetric matrix *Q* from the SBP community using (7) in each element. It is given by

$$Q = \begin{pmatrix} -\frac{1}{2} &  1 &  -\frac{1}{2} \\ -1 &  0 &  1 \\ \frac{1}{2} &  -1 &  \frac{1}{2} \end{pmatrix} $$

where $\textbf{u}= (u_j,\bar{u}_{j+1/2},u_{j+1})^T$. By direct calculation, (30) is naturally fulfilled with $B=\operatorname {diag}(-1, 0, 1)$ where we have exactness for constants in the underlying function space due to

$$D {\textbf {1}} =(\texttt{M}^{-1}Q){\textbf {1}}= \begin{pmatrix} -4 &  6 &  -2 \\ -1 &  0 &  1 \\ 2 &  -6 &  4 \end{pmatrix} \begin{pmatrix} 1 \\ 1 \\ 1 \end{pmatrix} ={\textbf {0}}. $$

This is essential for local conservation [29]. The PamPa scheme fulfills essential all the properties which has been proven for SBP schemes or techniques which can be applied inside one element. However, up to this point this is now only the simple dG formulation with a non-classical approximation space. As it is described in previous chapters, the PamPa scheme is not like this, but use continuous approximations instead of the dG version. Therefore, we have now to project back from the discontinuous vector space to the continuous one. In terms of PamPa, we can do this via upwinding or central (or combination of them) at the point values at the element interfaces, cf. Sect. 2.4. It means for example when upwinding or a central projection is considered. We have the following cases:

- **Upwind**: Drop the update of $u_j$:
  - if $a>0$: coming from the interval $I_{j+\frac{1}{2}}$ and keep the internal DoFs computed from dG.
  - If $a<0$, coming from the interval $I_{j-\frac{1}{2}}$ and keep the internal DoFs computed from dG.
- **Central**: Take the average of the left and right contribution at the point values.

Therefore, we have to adapt the point value update in each element concerning the specific framework used where the average value $\overline{u}_{{j+\frac{1}{2}}}$ is not touched. Please remember that our vector inside the element $I_{{j+\frac{1}{2}}}$ for a third order method looks like this $(u_j, \overline{u}_{{j+\frac{1}{2}}}, u_{j+1})^T$ whereas inside $I_{j+\frac{3}{2}}$ we have $(u_{j+1}, \overline{u}_{j+\frac{3}{2}}, u_{j+2})^T$. Focusing on the common element interface at $x_{j+1}$, we have a value for $u_{j+1}^-$ from the left calculated by the DoFs inside $I_{j+\frac{1}{2}}$ and alternatively one value from the right $u_{j+1}^{+}$. Using (9), we have for the update of $u_{j+1}^{-/+}$ the following

33 $$\begin{aligned} \frac{\mathrm d}{\mathrm dt}\begin{pmatrix} u_j \\ \overline{u}_{j+\frac{1}{2}}\\ {u_{j+1}^-} \\ {u_{j+1}^+}\\ \overline{u}_{j+\frac{3}{2}}\\ u_{j+2}\\ \end{pmatrix} +\frac{a}{\Delta x}\begin{pmatrix} 6\overline{u}_{j+\frac{1}{2}}-4u_j-2u_{j+1}^- \\ u_{j+1}^--u_j \\ 2u_j+4u_{j+1}^--6\overline{u}_{j+\frac{1}{2}}\\ 6\overline{u}_{j+\frac{3}{2}}-4u_{j+1}^+-2u_{j+1}^+ \\ u_{j+2}-u_{j+1}^+\\ 2u_{j+1}^++4u_{j+2}-6\overline{u}_{j+\frac{1}{2}}\end{pmatrix}=0. \end{aligned}$$

This would be the dG update where we focus on the two neighboring elements $I_{j+\frac{1}{2}}$ and $I_{j+\frac{3}{2}}$. In this context, the update of $u_{j+1}$ would be discontinuous over the element boundaries therefore we have to project back to the continuous approximation space. For simplicity reason later on, we select the central scheme. Here, we take the average to define the continuous representation over this cell interface $u_{j+1}= \frac{u_{j+1}^++u_{j+1}^-}{2}$. This yields to the update for $u_{j+1}$:

$$\begin{aligned} \frac{\mathrm d}{\mathrm dt} {u_{j+1}} +\frac{a}{\Delta x} \frac{1}{2} \left( 2u_j+4u_{j+1}-6\overline{u}_{j+\frac{1}{2}}+ 6\overline{u}_{j+\frac{3}{2}}-4u_{j+1}-2u_{j+2} \right) =0. \end{aligned}$$

resulting in

$$\begin{aligned} \frac{\mathrm d}{\mathrm dt} {u_{j+1}} +\frac{a}{\Delta x} \left( u_j-3\overline{u}_{j+\frac{1}{2}}+ 3\overline{u}_{j+\frac{3}{2}}-u_{j+2} \right) =0. \end{aligned}$$

This projection operator works on all interface values and therefore, we have instead of three updates in each cell, in PamPa we have only two to be considered. The updated derivative matrix has a banded structure and is given by

$$ \tilde{D}=\frac{1}{\Delta x} \begin{pmatrix} \ddots &  &  &  &  &  &  \\ -1 &  0 &  1 &  &  &  &  \\ 1 &  -3 &  0 &  3 &  -1 &  &  \\ \hline &  &  -1 &  0 &  1 &  &  \\ &  &  1 &  -3 &  0 &  3 &  -1 \\ \hline &  &  &  &  -1 &  0 &  1 \\ &  &  &  &  &  &  \ddots \end{pmatrix}, $$

where the embedded values represent one update inside an element. Here, we can easily derive a diagonal mass matrix via $\tilde{\textbf{M}}= \frac{\Delta x}{4} {{\,\textrm{diag}\,}}{(\cdots ,|3,1|,3,1,3,1, \cdots )}$. Again, the embedded version corresponds to the contribution in one element. Together we obtain $\tilde{\textbf{M}} \tilde{D}+ (\tilde{\textbf{M}} \tilde{D})^T=0$ which corresponds to an periodic SBP operator. Therefore, we can apply all the results of SBP in such context and obtain stability.

### Remark 4.1

Instead of using the central scheme, also upwind or other combination can be used. This would result in several different version of upwind SBP operators. Thanks to our proposition (2.6), energy stability can be proven in such context. However, the interesting question rises how the projection step from the discontinuous approximation space back to the continuous one effects the SBP properties. This is not only interesting in one-space dimension but in particular relevant in multi-dimension. Some works already exist concerning projection and extension of stability properties inside the SBP framework [31, 32]. Future work will be therefore considering the effect of the projection step on the SBP properties and the results emerged from this.

### Remark 4.2

During the ICOSAHOM 2025 conference in Montréal, a presentation was given on an ongoing work titled "Stability of the Active Flux Method in the Framework of Summation-by-Parts Operators" [33]. While we have taken note of this contribution, we would like to clarify that our work was developed independently. There has been no exchange of ideas with this group, which began prior to their presentation. Our investigation originates from a visit of one of the authors to SUSTech in early June 2025, during which valuable discussions were held with Professor Kailiang Wu, whose input we gratefully acknowledged. While both efforts concern the active flux method, the focus and context differ: our study is situated more within the finite element framework, in contrast to the finite difference setting emphasized in [33]. In particular, their work offers a contribution through a detailed analysis of mass matrix evaluation in the upwind active flux method. In contrast, our interest lies more in understanding the role and properties of the projection step from discontinuous to continuous approximation spaces, which we intend to explore further in future work. In our study, we did not pursue a in-depth stability analysis of the upwind SBP formulation, as Proposition 3.1 already ensures the relevant stability properties, rendering a detailed investigation unnecessary.

## Conclusions, Perspectives.

In this paper, we have shown a connection between the PamPa scheme and the dG formulation. One can see PamPa, for linear advection problem, as one step of dG followed by one projection onto the initial approximation step. There are several possible ways to project, we have shown two, and mainly more are certainly possible: what is the "best" one, in which sense? This reformulation of PamPa leads to a generalisation to non linear problems. This generalisation is not equivalent to the formulation contained in [19] and [14] for non linear problem. In [34] this reformulation of PamPa has been used to formulate in a much better way the discretisation of boundary conditions. We have also shown some intrinsic bound preserving properties of PamPa, linked to a generalisation of the Simpson formula. They have already been used in other publications to construct provable bound preserving schemes, and we also note that other solutions to the same problem are also available in the literature. Last, in one dimension, we have shown a SBP property of PamPa. One can imagine that a generalisation of this to multiD would provide an answer to the first question above: what is the “best” projection.

## Data Availability

Enquiries about data availability should be directed to the authors.

## VEM Approximation: Basic Facts.

Any basis of $V_k(P)$ is virtual, meaning that it is not explicitly computed in closed form. Consequently, the evaluation of $v_h(\textbf{x})$ at some $\textbf{x}\in P$ it is not straightforward. One way to proceed would be to evaluate $\pi (v_h)$, that is the $L^2$ projection of $v_h$ on $\mathbb {P}^k(P)$. Since we want to do it only using the degrees of freedom, it turns out that this is impossible in practice. But, as shown in [21, 22], it is possible to define a space $W_k(P)$ for which computing the $L^2$ projection is feasible. It is constructed from $V_k(P)$ in two steps. First, we consider the approximation space $\widetilde{V}_k(P)$ given by

$$\begin{aligned} \widetilde{V}_k(P)=\{ v_h, v_h \text { is continuous on }\partial P \text { and } (v_h)_{\partial P}\in \mathbb {P}^k(\partial P); \text { and }\Delta v_h\in \mathbb {P}^{k-2}(P)\}. \end{aligned}$$

For $p\in \mathbb {N}$, let $M^\star _p(P)$ be the vector space generated by the scaled monomial of degree *p* exactly,

$$\begin{aligned} m(\textbf{x})\in M^\star _p(P), \qquad m(\textbf{x})=\sum _{{\boldsymbol{\alpha }}, |{\boldsymbol{\alpha }}|=p} \beta _{{\boldsymbol{\alpha }}} m_{{\boldsymbol{\alpha }}}(\textbf{x}). \end{aligned}$$

Then, we consider $W_k(P)$, which is the subspace of $\widetilde{V}_k(P)$ defined by

$$\begin{aligned} W_k(P)=\{ w_h\in \widetilde{V}_k(P), \langle w_h-\pi ^\nabla w_h,q\rangle =0, \quad \forall q\in M^\star _{k-2}(P)\cup M^\star _k(P)\}. \end{aligned}$$

In [22], it is shown that $\dim V_k(P)=\dim W_k(P)$.

This approximation space is defined as follows. $w_h\in W_k(P)$ if and only if

1. $w_h$ is a polynomial of degree *k* on each edge *e* of *P*, that is $(w_h)_{|e}\in \mathbb {P}^k(e)$,
2. $w_h$ is continuous on $\partial P$,
3. $$\Delta w_h\in \mathbb {P}^{k-2}(P)$$,
4. $$\int _{P} w_h m_{{\boldsymbol{\alpha }}} \; \textrm{d}\textbf{x}=\int _{P} \pi ^\nabla w_h m_{{\boldsymbol{\alpha }}} \; \textrm{d}\textbf{x}$$ for $$|{\boldsymbol{\alpha }}|=k-1, k$$.

The degrees of freedom are the same as in $V_k(P)$:

1. The value of $w_h$ on the vertices of *P*,
2. On each edge of *P*, the value of $v_k$ at the $k-1$ internal points of the $k+1$ Gauss–Lobatto points on this edge,
3. The moments up to order $k-2$ of $w_h$ in *P*,
  $$\begin{aligned} m_{{\boldsymbol{\alpha }}}(w_h):=\dfrac{1}{\vert P\vert }\int _{P} w_h m_{{\boldsymbol{\alpha }}}\; d\textbf{x}, \qquad |{\boldsymbol{\alpha }}|\le k-2. \end{aligned}$$

The $L^2$ projection of $w_h$ is computable. For any ${\boldsymbol{\beta }}$, if $\pi ^0(w_h)=\sum _{{\boldsymbol{\alpha }}, |{\boldsymbol{\alpha }}|\le k} s_{{\boldsymbol{\alpha }}} m_{{\boldsymbol{\alpha }}}$, we have

$$\begin{aligned} \langle \pi ^0(w_h), m_{{\boldsymbol{\beta }}}\rangle =\sum _{{\boldsymbol{\alpha }}, |{\boldsymbol{\alpha }}|\le k} s_{{\boldsymbol{\alpha }}} \langle m_{{\boldsymbol{\beta }}}, m_{{\boldsymbol{\alpha }}}\rangle =\langle w_h, m_{{\boldsymbol{\beta }}}\rangle . \end{aligned}$$

The left hand side is computable since the inner product $\langle m_{{\boldsymbol{\beta }}}, m_{{\boldsymbol{\alpha }}}\rangle$ only involves monomials. We need to look at the right hand side. If $|{\boldsymbol{\alpha }}|\le k-2$, $\langle w_h, m_{{\boldsymbol{\beta }}}\rangle =|P| m_{{\boldsymbol{\beta }}}(w_h)$ and if $|{\boldsymbol{\alpha }}|=k-1$ or *k*, we have

$$\begin{aligned} \langle w_h, m_{{\boldsymbol{\beta }}}\rangle =\langle \pi ^\nabla w_h, m_{{\boldsymbol{\alpha }}}\rangle , \end{aligned}$$

which is computable from the degrees of freedom only.

We note that if $w_h\in W_k(P)$, then $m_{{\boldsymbol{\alpha }}}(\pi ^0 w_h)=m_{{\boldsymbol{\alpha }}}(w_h)$. Indeed

$$\begin{aligned} m_{{\boldsymbol{\alpha }}}(\pi ^0 w_h)=\frac{1}{|P|} \langle \pi ^0 w_h ,m_{{\boldsymbol{\alpha }}} \rangle =\frac{1}{|P|} \langle w_h ,m_{{\boldsymbol{\alpha }}} \rangle \end{aligned}$$

by construction. This is not true for $\pi ^\nabla$ in $V_k(P)$. However, for $k\le 2$, $V_k(P)=W_k(P)$

The last remark is that, since $\mathbb {P}^k(P)\subset V_k(P)$ and $\mathbb {P}^k(P)\subset W_k(P)$, the projections of $C^{k+1}(P)$ onto $V_k(P)$ and $W_k(P)$ defined by the degrees of freedom is $k+1$-th order accurate.

We recall how to approximate the mass matrix. We have for any *i* and *j* that represent the index of any degree of freedom,

$$\int _P\varphi _i\varphi _j=\big (\pi \varphi _i,\pi \varphi _j\big )+ \big ({\textbf {Id}}-\pi )\varphi _i, ({\textbf {Id}}-\pi )\varphi _j)$$

because for any $u\in L^2(K)$, we have,v

$$\begin{aligned} \big ( ({\textbf {Id}}-\pi )u,\pi v\big )=0 \end{aligned}$$

The term

$$\begin{aligned} \big (\pi \varphi _i,\pi \varphi _j\big ) \end{aligned}$$

can be computed exactly, while we approximated

$$\big ( \big ({\textbf {Id}}-\pi )\varphi _i, ({\textbf {Id}}-\pi )\varphi _j)\approx \vert P\vert \sum _{r=1}^{N_{dofs}} DOF_r\big ( ({\textbf {Id}}-\pi )\varphi _i\big ) DOF_r\big ( ({\textbf {Id}}-\pi )\varphi _j\big )$$

The sum of the two matrices is invertible.

## Footnotes

1 Following the finite element convention, we use the subscript ${\textrm{h}}$ to indicate a finite element approximation of a variable.

2 This can occur for example in the case where $\textbf{f}(\textbf{u})=\textbf{a}\textbf{u}$, $\sigma$ is the mid point of an edge parallel to $\textbf{a}$. In that case up-winding makes no sense and the weight $\tilde{\omega }_{\boldsymbol{\sigma }}$ is equal to $\tfrac{1}{2}$.
